# On the Origins of Enzymes: Phosphate-Binding Polypeptides Mediate Phosphoryl Transfer to Synthesize Adenosine Triphosphate

**DOI:** 10.1021/jacs.2c08636

**Published:** 2023-03-23

**Authors:** Pratik Vyas, Sergey Malitsky, Maxim Itkin, Dan S. Tawfik

**Affiliations:** † Department of Biomolecular Sciences, Weizmann Institute of Science, Rehovot 7610001, Israel; ‡ Department of Life Sciences Core Facilities, Weizmann Institute of Science, Rehovot 7610001, Israel

## Abstract

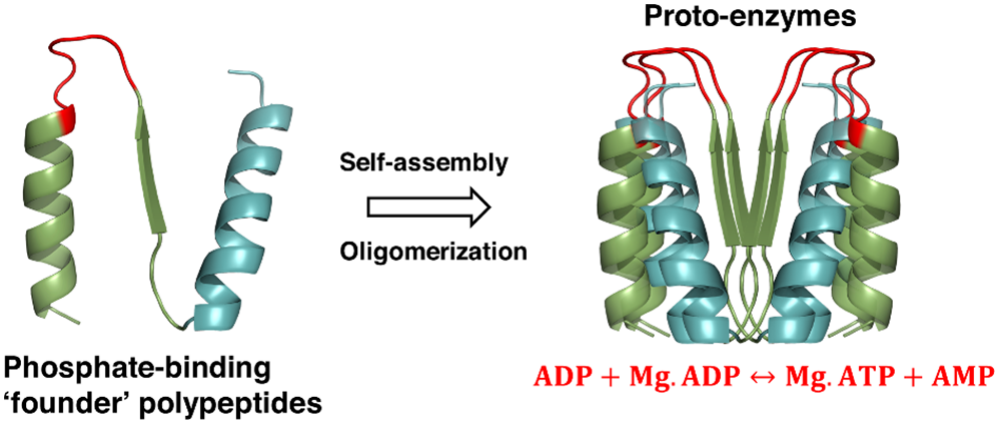

Reactions involving the transfer of a phosphoryl (−PO_3_^2–^) group are fundamental to cellular metabolism. These reactions are catalyzed by enzymes, often large and complex, belonging to the phosphate-binding loop (P-loop) nucleoside triphosphatase (NTPase) superfamily. Due to their critical importance in life, it is reasonable to assume that phosphoryl-transfer reactions were also crucial in the pre-LUCA (last universal common ancestor) world and mediated by precursors that were simpler, in terms of their sequence and structure, relative to their modern-day enzyme counterparts. Here, we demonstrate that short phosphate-binding polypeptides (∼50 residues) comprising a single, ancestrally inferred, P-loop or Walker A motif mediate the reversible transfer of a phosphoryl group between two adenosine diphosphate molecules to synthesize adenosine triphosphate and adenosine monophosphate. This activity, although rudimentary, bears resemblance to that of adenylate kinase (a P-loop NTPase enzyme). The polypeptides, dubbed as “P-loop prototypes”, thus relate to contemporary P-loop NTPases in terms of their sequence and function, and yet, given their simplicity, serve as plausible representatives of the early “founder enzymes” involved in proto-metabolic pathways.

## Introduction

In phosphoryl-transfer reactions, a phosphoryl group (−PO_3_^2–^) is transferred from a phosphate ester or an anhydride to a nucleophile. These reactions have some of the slowest uncatalyzed rates in biology and therefore demand significant rate acceleration from biological catalysts.^1,2^ In living cells, phosphoryl-transfer reactions are primarily catalyzed by enzymes such as kinases, adenosine triphosphate (ATP)/guanosine triphosphatases (GTPases), and phosphatases that belong to the phosphate-binding loop (P-loop) nucleoside triphosphatase (NTPase) superfamily.^3−6^ The P-loop NTPases are one of the most abundant, functionally diverse, superfamilies and implicated in essential life processes such as protein synthesis and maintenance, RNA/DNA modeling, ATP synthesis, and cellular signaling and metabolism.^1−5,7^ These enzymes are often large and complex, and their precise functioning depends on a finely tuned coordination among their subunits and cellular architecture.^8,9^ ATP synthases, for instance, are multi-subunit, 600 kDa molecular turbines that utilize the proton-motive force across a cellular membrane, generated by oxidation of a reductant, to drive ATP synthesis.^9^ However, in contrast to the structural and functional complexity is the postulate that modern proteins emerged by duplication, fusion, and self-assembly of “seed” (ancient) peptide fragments^10,11^ and by random polymerization of prebiotic amino acids.^12,13^ Therefore, it is reasonable that complex machines such ATP synthases and other enzymes involved in phosphoryl transfer must have emerged and evolved from simpler, seeding, progenitors that were nonetheless able to carry out the core catalytic phosphoryl-transfer reaction. Our goal is to identify, experimentally reconstruct, and functionally validate the so-called “seed” peptides to understand how complex proteins, and their function, emerged and evolved.

Although present-day proteins have undergone significant sequence and structural alterations throughout evolution, the seed fragments have remained unchanged owing to their functional importance.^11^ A classical instance is the Walker A (P-loop) motif, a glycine-rich loop, defined as GxxxxGK(T/S) or GxxGxGK, that underlies all the proteins belonging to the P-loop NTPase superfamily.^3^ Structurally, the core NTPase domain is composed of repeating β–α elements connected by short “bottom” loops and long and flexible “top” loops that harbor active site residues.^4,8^ The P-loop motif is nestled, invariably, in the first β–loop-α element and, accordingly, is dubbed as the “β1–P-loop-α1” motif or more generally as the “β-P-loop-α” motif. In present-day P-loop NTPases, the P-loop binds phosphorylated ribonucleosides and catalyzes the transfer of the phosphoryl group with the help of other auxiliary residues.^3,14,15^ In addition to the P-loop, nucleotide binding is also conferred by a short stretch of abiotic amino acids, via backbone amides and side-chain interactions, connecting the P-loop to the adjoining helix in the “β-P-loop-α” motif.^16,17^ This observation is also in line with the widely accepted notion that P-loop NTPases emerged at early stages of protein evolution, possibly at the interface of the RNA and RNA–protein worlds^11,18^ and that binding to phosphate ligands, such as nucleotides and nucleotide cofactors, is a critical ancient function.^5,11,18−22^ Accordingly, the β-(P-loop)-α motif has been proposed to be the “seed segment” from which extant P-loop NTPases have emerged.^21,23−25^

**Figure 1:**
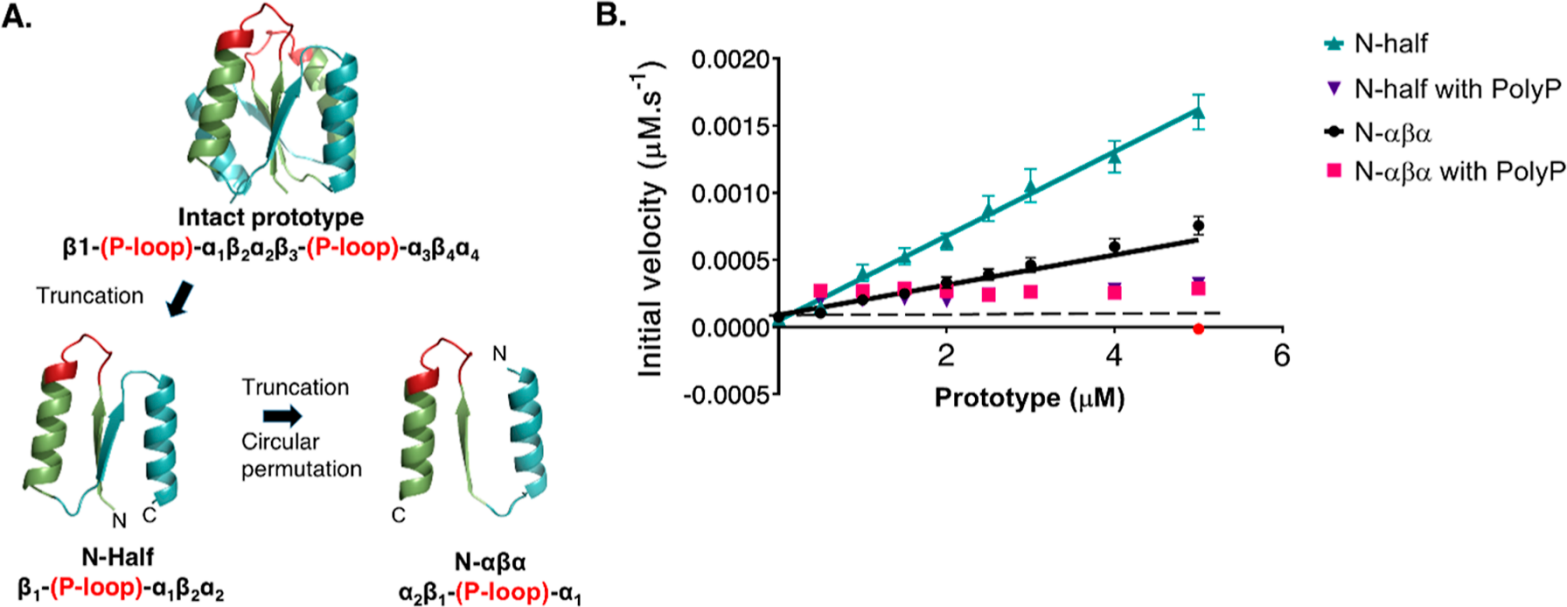
P-loop prototypes and luciferase assay to detect ATP synthesis. (A)Representative P-loop prototypes used for this study. The “intact” prototype contains two copies of the β-(P-loop)-α motif connected by scaffolding β-(loop)-α elements [topology: β1-(P-loop)-α1−β2-(loop)-α2−β3-(P-loop)-α3−β4-(loop)-α4.^26^ Short prototypes “N-half” and “N-αβα” were constructed from the “intact” prototype by truncation and circular permutation.^26^ The structural models depict the ancestrally inferred β1 strand and α1 helix in green and the connecting P-loop in red. The scaffolding β strands, α helices, and connecting loops are shown in cyan. The descriptor below each prototype indicates the strand topology and the order of secondary structural elements. The prototypes have a propensity to oligomerize;^26^ therefore, the monomeric models shown here are only schematic descriptions. The models of P-loop prototypes are adapted using PyMOL (pymol.org) with permission from ref (26). Copyright 2021, Proceedings of the National Academy of Sciences of the United States of America. Sequences of prototypes are listed in Supporting Information Table S2. (B) ATP-synthesis activity of P-loop prototypes. Luciferase assay showing a linear increase in ATP synthesis (expressed as μM of ATP synthesized per second) with the increasing concentration of prototypes. N-half with ADP (turquoise triangles); N-αβα with ADP (black circles); N-half with ADP and PolyP (violet triangles); and N-αβα with ADP and PolyP (pink squares). The black dotted line indicates the background luminescence from 1 mM ADP (generally equivalent to 0.2–0.3 μM ATP). The red circle on the *x*-axis indicates background luminescence from 5 μM prototypes. Reactions were carried out in the presence of 1 mM ADP and 0.5 mM MgCl_2_ with and without 0.5 mM PolyP at 37 °C for 1 h (see the “Materials and Methods” section). Error bars represent the standard error of mean (SEM) from four to eight independent experiments.

Our group has previously described βα proteins, dubbed as “P-loop prototypes”, composed of the ancestrally inferred β-(P-loop)-α motif grafted onto a rudimentary scaffold that mimics the P-loop NTPase core.^23,26^ The largest “intact” prototype (110 residues) contains two copies of the β-(P-loop)-α motif connected by scaffolding β-(loop)-α elements [intact prototype: **β1-(P-loop)-α1**–β2-(loop)-α2–**β3-(P-loop)-α3**–β4-(loop)-α4)] (Figure 1A).^23,26^ In a succeeding study, we were able to shorten the structural context of the intact prototype down to 40–60 residues.^26^ These short prototypes have low complexity, i.e., they do not contain any of the active sites and auxiliary residues of contemporary P-loop NTPases^23,26^ and are composed mostly of abiotic amino acids.^26^ Despite their simplicity, these polypeptides retain the ability to bind phosphorylated ligands such as ATP/GTP/adenosine diphosphate (ADP)/adenosine monophosphate (AMP)^23,26^ and bind most avidly to inorganic phosphoanhydrides, such as tri- or poly-phosphates^26^ (the proposed primordial energy precursors of dNTPs^27^). In addition, the shorter prototypes even show elaborate nucleic acid remodeling functions such as DNA unwinding and strand exchange.^26^

Considering the ability to bind various phosphorylated ligands, we were encouraged to investigate if these short, yet functional, prototypes could mediate the transfer of a phosphoryl group between the former. We were further guided by the observation that enzymes such as phosphotransferases, adenylate kinases, and other nucleotide kinases are widely represented in the last universal common ancestor^5^ and thus, phosphoryl-transfer reactions were also likely to be crucial in the primordial world. While the largest prototype, i.e., “intact” prototype (∼110 residues), appears to mediate phosphoryl transfer by weakly hydrolyzing ATP, ADP, and AMP under specific conditions,^23^ hydrolysis is of little evolutionary utility without a parallel ability to synthesize NTPs. Therefore, to explore the biotic origins of metabolic energy sources, we asked if P-loop polypeptides could synthesize NTPs.

## Results and Discussion

### P-Loop Prototypes Mediate Phosphoryl Transfer to Synthesize ATP

As representatives to test for phosphoryl-transfer activity, we used two short prototypes from our previous report:^26^ (1) N-half: the N-terminal half of the intact prototype and (2) N-αβα: a circular permutation construct consisting of only the ancestral β-(P-loop)-α motif and an additional helix that promotes solubility (Figure 1A). While most of the short prototypes described in our previous report were functional for binding to phosphate ligands, the prototypes for this study were chosen primarily for their better expression yields and purity.^26^

Guided by the observation that P-loop prototypes bind avidly to inorganic polyphosphates,^26^ we asked if the prototypes could transfer a phosphoryl group from inorganic polyphosphates to ADP and synthesize ATP. This activity resembles that of polyphosphate kinases, an ancient class of bacterial enzymes, that reversibly transfer the terminal phosphate group from inorganic polyphosphate to β-phosphate of ADP to synthesize ATP.^28^ To assay for ATP synthesis, we used a conventional bioluminescence assay that employs firefly luciferase to generate light in the presence of its substrate (luciferin), oxygen, Mg^+2^, and ATP.^29^ A typical experimental setup involved incubating the prototypes with inorganic polyphosphate (PolyP; 25-mer) and ADP. ATP, if generated in the reaction, was detected by measuring luminescence upon the addition of a luciferase premix (containing luciferase, luciferin, and MgCl_2_) and quantified using a standard curve calculated by measuring, in parallel, luminescence from “ATP-only” controls (see the “Materials and Methods” section; Supporting Information Figure S1). To begin with, prototypes (0 to 5 μM) were incubated with presumed saturating concentrations of ADP (1 mM) and PolyP (0.5 mM) for 1 h under optimized reaction conditions: in tricine buffer (pH 7.6) containing 0.5 mM MgCl_2_ at 37 °C (see the “Supporting Information Materials and Methods” section for details on the optimization of the reaction conditions). Figure 1B shows that P-loop prototypes synthesize ATP from ADP itself (without PolyP; turquoise and black lines) and this activity, expressed as μM of ATP produced per second, increases linearly with increasing concentrations of the prototypes. The larger, “intact”, prototype also mediates ATP-synthesis activity (Supporting Information Figure S2) that is comparable to that of shorter prototypes. The shorter prototypes, however, are simpler relative to the “intact” construct (in terms of sequence complexity and number of residues) and, thus, more plausible representatives of early enzymes. Therefore, we chose to characterize the phosphoryl-transfer (used interchangeably with ATP-synthesis) activity of the shorter constructs.

### Inorganic Polyphosphates Appear to Inhibit ATP-Synthesis Activity

For the reactions containing ADP and PolyP (25-mer), the ATP levels are barely above the background luminescence from the 1 mM ADP control (black dotted line; Figure 1B). Further, we do not observe any increase in activity with the increasing concentration of prototypes (Figure 1B). Previously, we showed that P-loop prototypes demonstrate avid binding to inorganic polyphosphates.^26^ This avidity is likely due to multiple phosphate groups contributed by long-chain inorganic polyphosphates and due to the overall positive charge on the prototypes, especially in the P-loop region (Supporting Information Table S2 and Figure S3). Consequently, PolyP (25-mer) inhibits the reactions even at 1 μM concentration (despite having 1 mM ADP in the reaction) (Supporting Information Figure S4). It is also possible that 25-mer PolyP outcompetes ADP for all available binding sites on the prototypes. Therefore, we tested if the prototypes can mediate phosphoryl transfer from inorganic triphosphates to ADP. Inorganic triphosphates also appear to inhibit the reaction at high concentrations (100–500 μM; Supporting Information Figure S4). In the reactions with low triphosphate concentrations (1–10 μM), ATP synthesized is, at best, comparable to the reactions without triphosphates (Supporting Information Figure S4). We cannot entirely rule out the possibility that a fraction of ATP synthesized in the reaction may be a consequence of phosphoryl transfer from triphosphate to ADP. However, this is only possible at very low triphosphate concentrations that do not show significant inhibition. Crucially, the presence of ADP in the reactions makes such an analysis extremely challenging, given that the prototypes can synthesize ATP from ADP alone. Nonetheless, the ability of the prototypes to mediate the transfer of a phosphoryl group, be it from inorganic polyphosphates or ADP, is significant in the context of the primordial world (see the “General Discussion” section) and was characterized further.

### ATP-Synthesis Activity Is Dependent on Divalent Metal Ions

Next, we tested the effect of various metal ions on the ATP-synthesis activity of P-loop polypeptides. The presence of divalent cations such as Mg^+2^, Mn^+2^, and, to a lesser degree, Ca^+2^ is essential for the activity of prototypes (Supporting Information Figure S5). This dependency is unsurprising as divalent metal ions, foremost magnesium cations, are an obligatory requirement in catalyzing phosphoryl-transfer activity in contemporary P-loop NTPases^14,30,31^ (with notable exceptions; ref (32)). Titrating with varying concentrations of MgCl_2_, at constant ADP (1 mM) and prototype (10 μM) concentrations, showed that the activity plateaued around 0.5 mM MgCl_2_ (Supporting Information Figure S5).

To deduce the reactive species for ATP-synthesis activity, we calculated the fraction of bound (Mg·ADP) and free ADP in the reaction, using the known dissociation constant of the Mg·ADP complex (see the “Supporting Information Materials and Methods” section). The prototypes demonstrate the maximal activity when the reaction concentrations of Mg·ADP and ADP are approximately equal (i.e., at ∼0.5–1 mM MgCl_2_) (Supporting Information Materials and Methods), allowing us to speculate that both Mg^+2^-bound and unbound ADP are required for ATP synthesis. Presumably, for phosphoryl transfer to occur, both Mg·ADP and ADP should bind to two different sites in the ternary complex, in the correct geometry, with their phosphates pointing toward each other. In line with our data, previous reports with similar kinetic trends (optimal activity at equal concentrations of Mg^+2^-bound and unbound ADP) have suggested that both Mg·ADP and ADP are the substrates for phosphoryl transfer enzymes.^33,34^

As more Mg·ADP is formed, with the increase in MgCl_2_ concentrations, there is progressive depletion of free ADP, and accordingly, we observe a decrease in activity (Supporting Information Materials and Methods and Figure S5). Yet, one may argue that activity is largely consistent despite depleted ADP levels. We note that the depletion of ADP is concomitant with the increase in free MgCl_2_ in the reaction that, in turn, may influence the activity. While one Mg^+2^ ion is sufficient for phosphoryl-transfer activity, at high concentrations of free MgCl_2_ (generally higher than 1 mM), kinases can accommodate a second Mg^+2^ ion in the active site and mediate phosphoryl transfer via “two-metal catalysis”.^31^ The presence of additional Mg^+2^ ion can affect the structural and kinetic parameters of many kinases.^31,35−41^ Molecular dynamics calculations have shown that activation energy for phosphoryl transfer is lower for kinases when two Mg^+2^ ions are bound in the active site (ref (41) and other references therein). Further, the presence of an additional Mg^+2^ ion in the active site has been shown to lower the *K*_m_ of nucleotide substrates of kinases.^31^ Finally, the binding of two Mg^+2^ ions may also promote structural changes to the active sites of the prototypes, resulting in a rigid, closed conformation of the P-loop, facilitating better exclusion of water molecules, thereby improving catalytic properties, as has been demonstrated for other phosphoryl transfer enzymes.^39^ Overall, magnesium ions are crucial for ATP synthesis and the prototypes mediate optimal activity at a ∼1:1 ratio of Mg·ADP and ADP—the true substrates of the reaction.

Next, we assessed the effect of temperature and pH on the ATP-synthesis activity. The prototypes demonstrate comparable, maximal, activities at 37, 42, and 45 °C; whereas the pH titrations revealed that the activity is stable over a pH range of 6 to 9 (Supporting Information Figure S6A, B). Henceforth, the experiments shown in Figure 1B and all subsequent experiments were performed under optimized conditions: 0.5 mM MgCl_2_,_,_ 37 °C, and pH 7.6.

**Figure 2:**
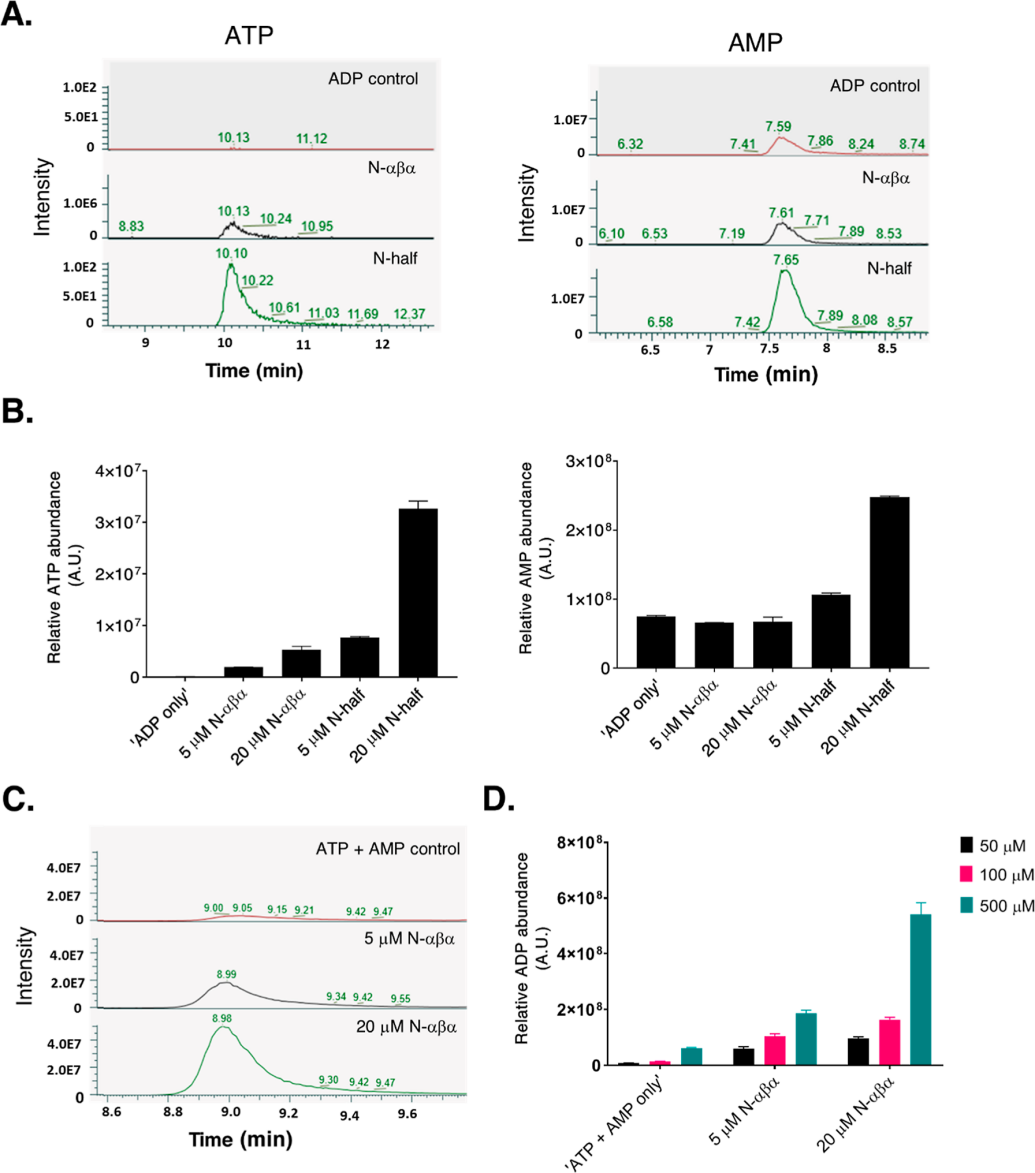
LC–MS analysis of phosphoryl-transfer activity of P-loop prototypes. (A) Representative extracted mass chromatograms depicting the intensity of ATP ([M – H]^−^, *m*/*z* = 505.9885) and AMP ([M – H]^−^, *m*/*z* = 347.06363) for test reactions containing 20 μM N-half and N-αβα prototypes, 1 mM ADP, and 0.5 mM MgCl_2_. “ADP control” reactions contain 1 mM ADP and 0.5 mM MgCl_2_. (B) Relative abundance corresponding to ATP and AMP (from A) analyzed by LC–MS*. Shown are bar plots for control (“ADP only”) and test samples (5 and 20 μM of prototypes with 1 mM ADP and 0.5 mM MgCl_2_). Error bars represent the standard deviation from three independent measurements. (C) Representative extracted mass chromatograms depicting the intensity of ADP ([M – H]^−^, *m*/*z* = 426.0221) for test reactions containing 5 and 20 μM N-αβα prototype, equimolar concentrations of ATP and AMP ([ATP] = [AMP] = 0.5 mM), and 0.5 mM MgCl_2_. ATP + AMP control reactions contain equimolar concentrations of ATP and AMP ([ATP] = [AMP] = 0.5 mM) and 0.5 mM MgCl_2_. (D) Relative abundance corresponding to ADP (from C) analyzed by LC–MS. Shown are bar plots for control (“ATP + AMP only”) and test samples [5 and 20 μM of N-αβα prototype incubated with varying equimolar concentrations of ATP and AMP: 50 μM (black bar); 100 μM (pink bar); and 500 μM (turquoise bar)]. Error bars represent the standard deviation from three independent measurements. All reactions were performed in 50 mM tricine buffer (pH 7.6) and incubated at 37 °C for 1 h. *The relative abundance values of charged species of ATP and AMP depicted in Figure 2B should not be compared to each other. These values should also not be considered as a proxy for the amount of the product produced in the reaction as they can vary for different ligands depending on their ionization differences.

To validate that the luminescence signal measured in the luciferase assays is due to ATP formation, the reaction mixes (prototypes + ADP + MgCl_2_) were analyzed qualitatively by liquid chromatography–mass spectrometry (LC–MS). The presence of ATP was detected in the reaction mixes of both N-half and N-αβα prototypes (Figure 2A). In line with the protein concentration-dependent increase in activity (Figure 1B), we observed a higher abundance of ATP with 20 μM N-half prototype, as compared to that with 5 μM (Figure 2B). In addition to ATP, we also detected AMP in the reaction mixes with the prototypes. The relatively high AMP background in the ADP-only samples could be due to the dissociation of a fraction of ADP to AMP and/or due to contaminant nucleotides in the commercial ADP batches (98% purity). Nonetheless, the AMP abundance for the “20 μM N-half” sample is significantly higher (*p* < 0.0001) than that for the “ADP-only” samples. This activity of P-loop prototypes resembles the activity of adenylate kinase enzymes (belonging to the P-loop NTPase class) that catalyze the reversible conversion of ADP to ATP and AMP.^42^ In line with this, the N-αβα prototype also mediates the conversion of ATP and AMP to ADP (Figure 2C, D). Thus, the LC–MS experiments provided independent verification of ATP-synthesis activity observed in the luciferase assays.

### P-Loop Prototypes Are Weak “Catalysts”

**Figure 3:**
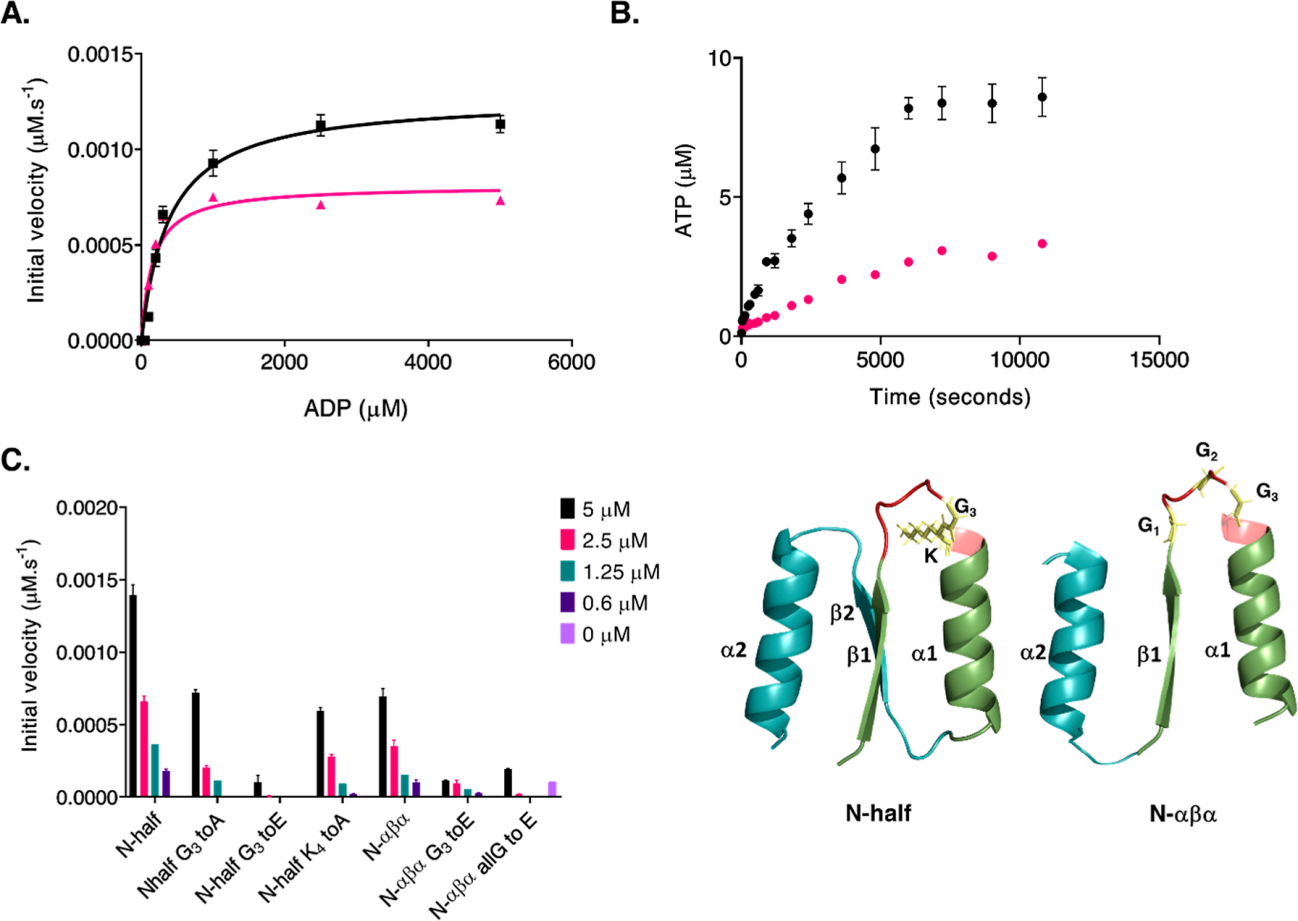
Kinetic parameters of ATP-synthesis activity. (A) Initial velocity of ATP synthesis as a function of ADP concentration. 4 μM prototypes (N-half, black curve and N-αβα, pink curve) were titrated with varying concentrations of ADP (50 to 5000 μM). Reactions were incubated for 1 h at 37 °C in tricine buffer (pH 7.6) with 0.5 mM MgCl_2._ Shown here are the initial velocities (expressed as μM ATP synthesized per second) for N-half and N-αβα prototypes at varying ADP concentrations. Data were fit to the standard Michaelis-Menton equation in GraphPad Prism 8.3.0. Error bars represent the SEM from four to eight independent measurements. (B) Time course analysis of ATP-synthesis activity. The N-half prototype (4 μM; black circles and 1 μM; pink circles) was incubated in the presence of saturating ADP concentrations (1 mM) in tricine buffer (pH 7.6) with 0.5 mM MgCl_2_. Luminescence was measured at varying time points, and ATP synthesized in the reaction was calculated using standard curves (see the “Materials and Methods” section). Error bars represent the SEM from three independent measurements. (C) ATP-synthesis activity of prototypes and P-loop mutants. Shown here are steady-state (1 h) values of ATP synthesis at varying concentrations of “wild-type” (N-half and N-αβα) and mutant prototypes. Reactions were carried out in the presence of 1 mM ADP and 0.5 mM MgCl_2_. Samples were incubated at 37 °C for 1 h. The “0 μM” (purple) bar indicates background luminescence from the ADP control sample (1 mM ADP + 0.5 mM MgCl_2_). The structural models of the N-half [topology: β1-(P-loop)-α1−β2-α2] and N-αβα prototypes [topology: α2−β1-(P-loop)-α1] are shown next to the bar diagram. The P-loop (G_1_xxG_2_xG_3_KT) connecting the β1 strand to α1 helix is colored red, and the mutated regions are shown as yellow sticks. Error bars represent the SEM from three to six independent measurements. The models of P-loop prototypes are adapted using PyMOL (pymol.org) with permission from ref (26). Copyright 2021, Proceedings of the National Academy of Sciences of the United States of America.

The experiments described so far were performed under a presumed saturating concentration of ADP (1 mM). Next, we measured steady-state kinetics of ATP synthesis at varying ADP concentrations. The apparent *K*_M_ of the N-half prototype (*K*_Mapp_) for ADP was 130 (±20) μM (Figure 3A pink fit curve and Table 1), whereas the N-αβα prototype demonstrated a lower ADP *K*_m_ of 67 (±18) μM (Figure 3A turquoise fit curve and Table 1). For both prototypes, however, we observed similar initial velocities: 0.001 μM ATP s^–1^ (N-half) and 0.0009 μM ATP s^–1^ (N-αβα) for 4 μM prototype (Table 1). By extension, both prototypes demonstrate comparable turnover numbers, i.e., apparent *k*_cat_ (*k*_cat_app__) values: 0.00026 s^–1^ (N-half) and 0.0002 s^–1^ (N-αβα), translating to approximately one turnover per hour (Table 1). For the end-point (one-hour incubation) measurements, ATP synthesis plateaus beyond 1 mM ADP concentration (Figure 3A). Next, a time course analysis revealed two peculiar observations. First, although the ATP formation increases beyond the hour mark, the reaction rates progressively decreased (Supporting Information Table S3), and second, the total product formation appears to plateau around the 7000 s (∼2 h) mark despite the presence of excess unused substrate (Figure 3B).

**Table 1:** Kinetic Parameters of ATP-Synthesis Activity of P-Loop Prototypes^a^

N-half prototype		N-αβα prototype	
*K*_Mapp_	130 (20) μM	*K*_Mapp_	67 (18) μM
*V*_max_app__	1.04 (0.01) × 10^–3^ μM ATP s^–1^ (i.e. 3.7 (0.3) μM ATP h^–1^)	*V*_max_app__	0.9 (0.1) × 10^–3^ μM ATP s^–1^ (i.e. 3.2 (0.4) μM ATP h–^1^)
*k*_cat_app__	2.6 (0.2) × 10^–4^ s^–1^ (i.e. 0.9 (0.1) h^–1^)	*k*_cat_app__	2.0 (0.3) × 10^–4^ s^–1^ (i.e. 0.8 (0.1) h^–1^)
*k*_cat___app__/*K*_Mapp_	2.5 (0.3) × 10^–6^ μM^–1^ s^–1^	*k*_cat___app__/*K*_Mapp_	2.9 (0.3) × 10^–6^ μM^–1^ s^–1^

a Reactions were carried out with 4 μM prototypes in the presence of 0.5 mM MgCl_2_ at 37 °C with varying concentrations of ADP (50 to 5000 μM) in 50 mM tricine buffer (pH 7.6). Samples were incubated for 1 h, and luminescence was measured as described in the Materials and Methods section. Values in parentheses represent the SEM from four to eight independent measurements.

To test if the rate of ATP synthesis slows down due to accumulating products, the reactions shown in Figure 3A were preincubated with varying concentrations of APPcP (a non-hydrolyzable analogue of ATP) and AMP. APPcP inhibits ATP synthesis with an apparent inhibitor constant (Ki_app_) of 80 (20) μM; however, the inhibitory effect is stronger with AMP (Ki_app_ = 10 (1) μM) (Supporting Information Figure S7). As observed in present-day adenylate kinases,^43^ AMP (and the second ADP molecule) is expected to bind to the prototypes at a secondary binding site (other than the P-loop). Thus, the progressively weaker reaction rates are could be due to AMP outcompeting the second ADP molecule due to stronger binding to the prototype. Nonetheless, a stoichiometric starting concentration of the N-αβα prototype (500 μM) demonstrates faster reaction rates and almost complete conversion of the substrate into the product (Supporting Information Figure S8) within the one-hour experimental time scale.

To assess if the product formation can be increased further (for the experiment shown in Figure 3B), we added an aliquot of the prototype to the reaction once it reaches a plateau. Injection of “fresh” protein increases ATP formation further until it plateaus again after two hours, necessitating the injection of an additional protein aliquot (Supporting Information Figure S9). Thus, the increase in product formation upon the addition of “fresh” protein suggests that “product inhibition” is unlikely to be the cause of loss in activity around the two hour mark.

In natural enzymes, H-bonding interactions via strategically positioned second-shell and third-shell residues, as well as long-range interactions, evolved over billions of years of evolution, restrain active sites in an optimally aligned rigid state, promoting substrate specificity and efficient catalysis.^44−48^ Such stabilizing interactions are likely to be absent in P-loop prototypes (or primordial enzymes) that have not been subjected to computational or directed evolution-based optimization for efficient phosphoryl transfer. Nuclear magnetic resonance analysis has shown that the β-(P-loop)-α region of the “intact” prototype exists in at least two conformations in its unliganded form.^23^ Therefore, it is reasonable that the “truncated”/shorter prototypes, lacking a stable core, may demonstrate even higher structural flexibility of the P-loop region. Conformational isomerism of the active site, involving a tryptophan rotamer flip, has been shown to limit the catalytic efficiency of the catalytic antibody *34E4*.^49^

Structural plasticity also manifests as oligomeric heterogeneity of P-loop polypeptides. For instance, the N-αβα prototype can self-assemble to form higher-order oligomers (10 to 30-mers^26^) that can change upon ligand binding.^26^ Foremost, oligomerization is fundamental to the functioning of P-loop prototypes^23,26^ (see the “General Discussion” section). Given this tendency to exist in multiple interchangeable states and the aforementioned lack of stabilizing interactions, it is plausible that during the reaction time course, the prototypes adopt an alternate “sub-state” (i.e., conformations, oligomers, rotamers, tautomers, or even single-atom changes such as protonation states) that is not productive for catalysis. To this end, we monitored changes, if any, in the oligomeric forms of prototypes using the mass photometry method.^50^ During the two-hour reaction time frame, the N-αβα prototype in its unliganded form retains the larger oligomeric forms (corresponding to a 10-mer species), in line with our previous results from native mass spectrometry.^26^ However, in the presence of ADP and Mg^+2^, the prototype adopts a smaller oligomeric form (Supporting Information Figure S10). This change in the oligomeric state may result in either loss of binding or binding of ADP in an incorrect orientation (i.e., a “futile encounter”), relative to the active site residues, which does not result in phosphoryl transfer. Non-productive (futile) encounters, wherein the enzyme adopts a sub-state that is not conducive for catalysis, have been reported for enzymes with weak and promiscuous activities.^47,51^

**Figure 4:**
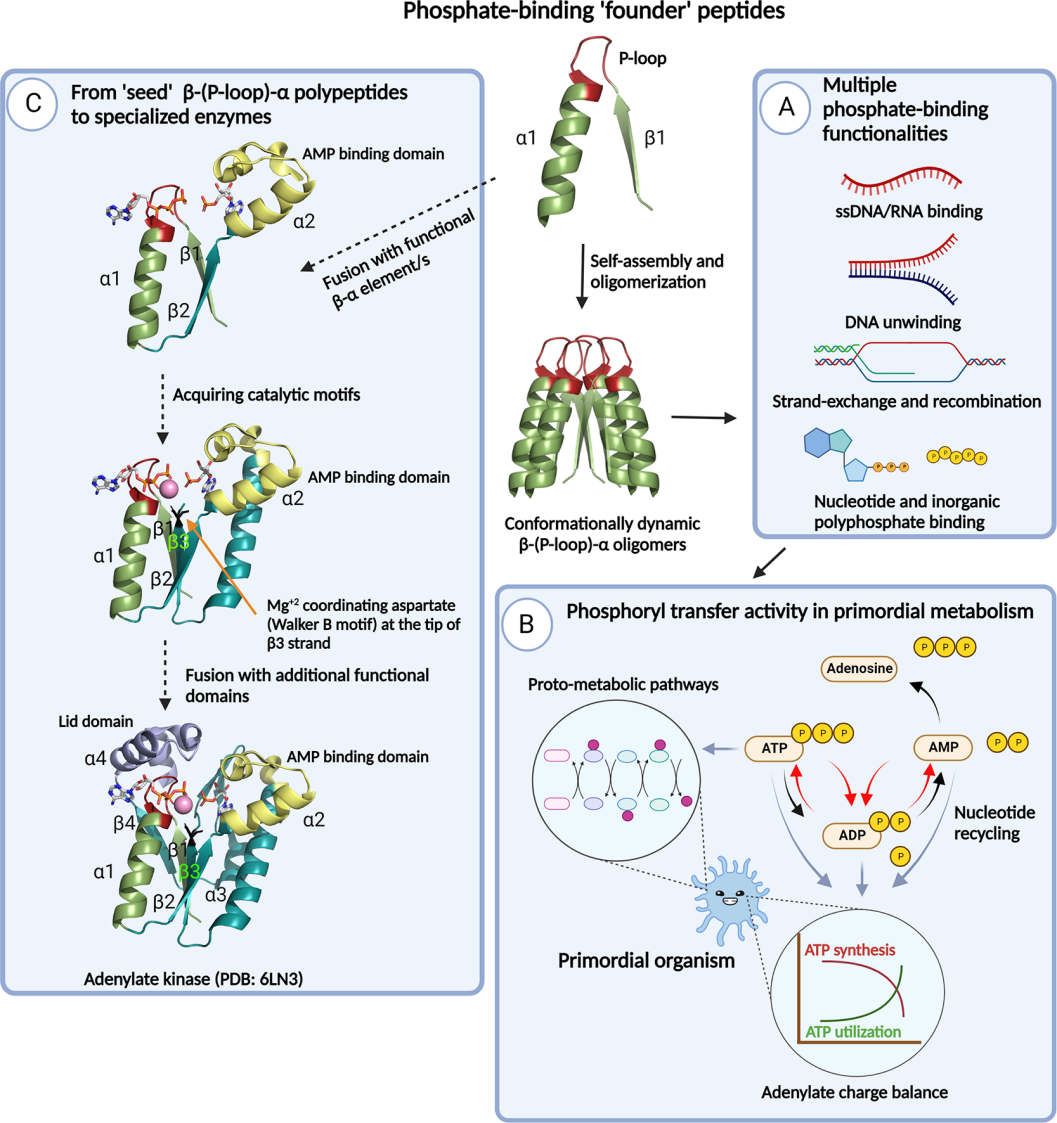
Evolutionary implications of P-loop prototypes and their phosphate-binding activity. The β1-(P-loop)-α1 seed motif self-assembles to form multiple higher-order oligomers that provide the structural volume necessary for binding and catalytic functions.^26^ Within these heterogeneous oligomeric forms, the β-(P-loop)-α motif may arrange in various conformational states,^23,26^ facilitating binding to various phosphorylated substrates and/or conferring the ability to bind to a given phosphate ligand using multiple binding modes. (A) Multifunctional phosphate binders. Oligomeric forms of β-(P-loop)-α demonstrate a multitude of phosphate-binding functions that are crucial in the RNA/RNA–protein world, such as binding to RNA/ssDNA and dsDNA,^23,26^ DNA-remodeling functions such as strand separation and exchange,^26^ binding to nucleotides,^23,26^ and, foremost, avid binding to inorganic polyphosphates,^26^ the presumed precursors of NTPs.^27^ (B) Phosphoryl-transfer activity of P-loop prototypes. The phosphoryl-transfer activity of P-loop polypeptides, which is a function of nucleotide binding, enables synthesis and recycling of nucleotides (red curved arrows), thereby diverting the reaction from its eventual fate, i.e., nucleosides plus phosphates (black curved arrows). This activity would have been crucial for maintaining the adenylate charge^61^ of a primordial organism and in fueling proto-metabolic pathways.^62−64^ (C) From the seed β-(P-loop)-α motif to extant enzymes. A hypothetical evolutionary trajectory (black dotted arrows) depicting the transition from seed peptides to adenylate kinase, a representative modern-day P-loop NTPase enzyme. A plausible trajectory comprises fusions with “βα” motifs such as the α2 helical segment (shown in yellow) that confers AMP binding capabilities to adenylate kinase enzymes. In line with this, we anticipate the binding to AMP or the second ADP to the α2 helix of the prototypes (Figures 1 and 3). Fusions with additional βα motifs, such as the β3 strand harboring the catalytic aspartate/glutamate (Walker B) residue and the “lid” domain (α4 helix), confer catalytic and rate-enhancement properties.^68^

High-resolution structural data may reveal the exact molecular details underlying the change in the oligomeric form and how this change translates to the apparent loss in activity. However, our attempts to this end using various approaches have not yielded convincing results, perhaps due to the “floppy” nature of prototypes. Overall, the structural plasticity of the β-(P-loop)-α region facilitates binding to multiple phosphorylated ligands^26,52^ (see the “General Discussion” section and Figure 4, panel A); however, this promiscuity comes at the expense of catalytic efficiency (Figure 3B). Nonetheless, this “floppiness” provides a basis for evolution to remodel primordial active sites with weak and promiscuous activities into efficient and specific enzyme functions.

### P-Loop Motif Mediates Phosphoryl-Transfer Activity

Our previous studies have shown that binding to phosphorylated ligands, be it ATP or DNA, is compromised when key residues of the P-loop (G_1_xxG_2_x**G**_**3**_**KT**) are altered.^23,26^ In the same vein, to confirm that the P-loop residues govern the ATP synthesis, we generated a set of prototype constructs with mutations to the conserved glycines and lysine of the P-loop (Figure 3C cartoon models). The alanine mutants (G_3_ to A and K to A), although weakened, retained some activity. It is noteworthy that alanine mutants have been reported to retain function in extant P-loop NTPases.^53^ Therefore, we tested potentially disruptive, glycine to glutamic acid, substitutions in the P-loop that were also used in our previous studies.^23,26^ Mutating the third glycine (G_3_ to E) and all glycines of the P-loop to glutamic acid (all G to E) abrogated the ATP-synthesis activity (Figure 3C). The loss of activity for G to E mutants was consistent, whereas the wild-type prototypes demonstrated ATP synthesis over multiple batches of expression and purification (Figures 1, 2, and 3A, B, and Supporting Information Figure S11). Overall, mutagenesis experiments confirm our previous finding that binding to phosphorylated ligands^23,26^ and, by extension, ATP synthesis is mediated by P-loop residues.

## General Discussion

That evolutionary relevant functions can emerge in seeding polypeptides is not obvious, and only a few experimental reconstructions to this end have been reported.^23,54−56^ Here, we demonstrate that polypeptides comprising a single, ancestrally inferred, β-(Ploop)-α motif mediates the reversible transfer of a phosphoryl group between ADP nucleotides to synthesize ATP and AMP.

The key to function in P-loop prototypes, or any primordial enzyme, is the aforementioned conformational and oligomeric heterogeneity.^23,26,57^ The propensity to oligomerize via self-assembly^26^ enables P-loop polypeptides to form a structural “framework” and an active-site pocket, by solvent exclusion, which is an essential requisite for binding and catalysis. Accordingly, a change in the oligomeric state, specifically a shift toward a smaller species, may result in a loss of activity (Supporting Information Figure S10). The conformational (including oligomeric) dynamism of the β-(P-loop)-α motif also allows the prototypes to employ more than one ligand binding mode, explaining why certain mutant prototypes (Figure 3; G_3_ to A and K_4_ to A mutants) retain some activity.

Described below are the evolutionary implications of phosphoryl-transfer activity of P-loop prototypes.

### P-Loop Prototypes and Primordial Metabolism

What evolutionary advantage would primordial peptides with a low turnover number provide? Although rate acceleration is a hallmark of enzymes, the role of enzymes also manifests in diverting the outcome of the reaction to yield a product different from that of a spontaneous reaction. Such diversions from the immediate thermodynamic and kinetic fates are critical for life and seen throughout the metabolism, certainly in biochemical pathways. While numerous reports have proven the possibility of prebiotic synthesis of nucleotides (ref (58) and the references therein, refs (59) and (60)), the eventual fate of these prebiotic nucleotides, in the absence of a primordial catalyst or a binder that stabilizes the ground states, would be nucleosides plus phosphates (black curved arrows in Figure 4, panel B). In such a scenario, primordial phosphate-binding polypeptides could channel ADP molecules to be converted into ATP and AMP (Figure 3A, B; red curved arrows in Figure 4, panel B), thus diverting the reaction’s eventual outcome (adenosines and phosphates). The AMP produced (along with ATP) could then be reconverted into ADP (red curved arrows in Figure 4, panel B). This recycling of adenine nucleotides would have been critical in maintaining the cellular energetic state—dubbed as “adenylate energy charge”^61^—of a primordial organism. Another consequence of phosphoryl-transfer activity of primordial peptides would be the enrichment of phosphorylated nucleosides or metabolites that could be readily accepted by other pre-existing primordial enzymes or peptides, thus fueling protometabolic networks.^62−64^

### From Multifunctional Phosphate Binders to Specialized Enzymes

Given the ubiquity of phosphate moieties in natural metabolites,^65^ proteins that bind phosphorylated ligands are highly abundant.^66^ Further, a systematic analysis has shown that phosphate binding, via short stretches of abiotic amino acids, is the earliest function of P-loop NTPases and other ancient protein lineages.^4,16,17^ Therefore, it is plausible that in a primordial world, new enzymes were recruited from an initial set of pre-existing, founding polypeptides that bound multiple forms of phosphates^26,67^ (Figure 4, panel A). These multifunctional polypeptides, demonstrating weak and diverse enzyme-like activities (refs (23) and (26) and this study), provide a basis for evolutionary fine-tuning and diversification into specialized functions. Along the evolutionary trajectory, fusion with additional domains would have conferred these seed polypeptides with enhanced functional and catalytic capabilities (Figure 4, black dotted arrows, panel C). For instance, present-day adenylate kinases possess “lid domains” that provide “closed” and “open” conformations for efficient phosphoryl transfer and the subsequent release of the products^68^ (Figure 4, panel C).

### From Nucleic Acid Remodelers to ATPases

Previously, we showed that P-loop prototypes have helicase-like functions such as strand separation and strand exchange.^26^ That these nucleic acid-remodeling prototypes also mediate phosphoryl transfer is consistent with the notion that F- and V-type ATPases evolved from ancestral RNA/DNA helicases.^9,69^ Therefore, in a primordial biotic world, substrate-level phosphorylation mediated by phosphate-binding polypeptides may have predated oxidative phosphorylation, which requires more advanced machineries such as a cellular membrane.^70^ In line with the Dayhoff’s hypothesis,^10^ such proto-peptides would have duplicated and fused to form repeats of β-(P-loop)-α motifs. The repeating units would have then diverged, wherein one β-(P-loop)-α retains nucleic acid binding function whereas the other binds to nucleotides and mediates phosphoryl transfer. A remnant of such duality is seen in an extant XPD helicase, wherein an ssDNA binding helicase C_2 domain is fused to the ATPase (DEAD) domain (ECOD domain ID: e6fwrA2).^26^

## Conclusions

In summary, despite their simplicity, the prototypes relate to modern-day enzymes involved in phosphoryl-transfer reactions and provide snapshots of how rudimentary enzymatic functions may have emerged in seeding polypeptides.

## Materials and Methods

### DNA and Cloning

Synthetic gene fragments coding P-loop prototypes were obtained from Twist Biosciences and were cloned into a pET29(+)b expression vector as described previously.^23^ Mutagenesis primers were obtained from IDT. Standard site-directed mutagenesis via restriction free cloning was used to generate mutant prototypes as described previously.^23^

### Protein Expression and Purification

P-loop prototypes have a C-terminal tag that is composed of a Trp residue for concentration determination by measuring absorbance at 280 nm (the prototypes are devoid of aromatic residues) followed by 6xHis for purification (DNA and amino acid sequences are provided in Supporting Information Tables S1 and S2). Following purification, the yield and purity of purified proteins was assessed by sodium dodecyl sulfate polyacrylamide gel electrophoresis (Supporting Information Figure S11). Typically, four peak elution fractions (total 7.5 mL) were pooled together and subjected to two rounds of dialysis (2 h at room temperature plus overnight at 4 °C) in a buffer containing 50 mM Tris and 100 mM NaCl (pH 8) to dialyze out the imidazole. The proteins generally precipitated during the dialysis step and required an osmolyte such as l-arginine to resolubilize the proteins. The samples were centrifuged; the protein pellet was collected and dissolved in a buffer containing 50 mM Tris (pH 8), 100 mM NaCl, and 1 M l-arginine (osmolyte to resolubilize the proteins). Purified prototypes, stored in this buffer at 4 °C at 100 to 200 μM concentrations, remained soluble and active for periods of 10–14 days.

### Luciferase Assay to Detect ATP Synthesis

The standardization of reaction conditions to determine the correct metal salt, temperature, and pH for activity is described in the Supporting Information Materials and Methods section. To test the potential of P-loop prototypes to synthesize ATP (Figure 1B), P-loop prototypes (0.5 to 5 μM) were incubated with 1 mM ADP in 50 mM tricine buffer (pH 7.6) with or without 0.5 mM PolyP. For ADP *K*_m_ measurements (Figure 3A), prototypes at a fixed concentration (4 μM), within the linear concentration range in Figure 1B, were incubated with varying ADP concentrations (50 to 5000 μM). All reactions were set up in 100 μL volumes in 96-well plates. Reactions were “initiated” by adding 0.5 mM MgCl_2_ and then incubated at 37 °C for 1 h. After 1 h incubation, 30 μL of the test reaction was transferred to 96-well flat white plates (Nunc), to which 30 μL of the luciferase premix (3.2 μM luciferase, 370 μM luciferin, and 10 mM MgCl_2_ in 50 mM tricine, pH 7.6) was added using a multi-channel pipette. Luminescence was measured in duplicates using a Tecan Infinite M-Plex plate reader with the “automatic attenuation” setting and 100 ms integration time. Background luminescence from “ADP only” control reactions (1 mM ADP + 0.5 mM MgCl_2_) and “protein only” reactions (5 μM prototypes without ADP and MgCl_2_) are shown in Figure 1B. Background luminescence corresponding to each “ADP only” sample (50 to 5000 μM) was measured and subtracted from luminescence from the respective test reactions. In parallel, luminescence from ATP controls (0.3 to 20 μM ATP with 1 mM ADP and 0.5 mM MgCl_2_) was measured using the same luciferase premix that was used for test reactions. Accordingly, an ATP standard curve was generated for each experiment (Supporting Information Figure S1) to quantify the ATP produced in the test reaction.

For time course analysis of ATP synthesis (Figure 3B), the N-half prototype (4 μM) was incubated with 1 mM ADP (or 0.25 mM ADP) in 800 μL reaction volume. The test reaction was incubated on a block thermostat to allow the reaction temperature to reach 37 °C. Meanwhile, luminescence from ATP dilutions (containing 1 mM ADP and 0.5 mM MgCl_2_) was measured, and a standard curve was generated (as described above). Once the reaction temperature for test reactions reached 37 °C (usually after 5–7 minutes), 30 μL of the test reaction was transferred to a 96-well white plate, and “time-zero” (i.e., without MgCl_2_) luminescence was measured by adding 30 μL of the luciferase premix. To “initiate” the reaction, MgCl_2_ was added to the reaction at a final concentration of 0.5 mM, and the reaction was mixed twice (by pipetting). 30 μL of the test reaction was transferred immediately to a 96-well plate, and luminescence was measured by adding 30 μL of the luciferase premix. The time lag between the addition of MgCl_2_ and the first measurement was 30 s. Likewise, luminescence was measured at varying time points for three hours. The luciferase premix, kept in dark (on ice), remained stable during the course of the experiment (Supporting Information Figure S12).

### LC–MS Analysis

For detection of ATP, P-loop prototypes (5 and 20 μM) were incubated with 1 mM ADP and 0.5 mM MgCl_2_ in tricine buffer (pH 7.6) in 500 μL reaction volumes, at 37 °C for 1 h. For detection of ADP, the N-αβα prototype (5 and 20 μM) was incubated with equimolar ratios (50:50, 100:100, and 500:500 μM) of ATP and AMP with 0.5 mM MgCl_2_ in tricine buffer (pH 7.6) in 500 μL reaction volumes, at 37 °C for 1 h. Proteins were filtered out using centrifugal filters with 3.5 kDa cutoff, and the flow-through was lyophilized.

a. Metabolite extraction: the lyophilized samples were reconstituted with 120 μL of a pre-cooled (−20 °C) homogeneous methanol (hypergrade, Merck): DDW (50:50, v/v) mixture. The tubes were vortexed for 15 s and then sonicated for 30 min in an ice-cold sonication bath (briefly vortexed every 10 min) and centrifuged at max speed at 4 °C. The supernatant was moved to a new Eppendorf tube and centrifuged again. Finally, 70 μL of the supernatant was transferred to the injection vials.
b. LC–MS polar metabolite analysis: metabolic profiling of samples was done as described by Zheng et al.^71^ with minor modifications described below. Briefly, analysis was performed using an Acquity I class UPLC system combined with a mass spectrometer Q Exactive Plus Orbitrap (Thermo Fisher Scientific), which was operated in a negative ionization mode. The LC separation was done using the SeQuant Zic-pHilic (150 mm × 2.1 mm) with the SeQuant guard column (20 mm × 2.1 mm) (Merck). Mobile phase B was acetonitrile (hypergrade, Merck) and mobile phase A was 20 mM ammonium carbonate with 0.1% ammonia hydroxide in DDW: acetonitrile (hypergrade, Merck) (80:20, v/v). The flow rate was set to 200 μL min^–1^, and the gradient was set as follows: 0–2 min 75% of B, 14 min 25% of B, 18 min 25% of B, 19 min 75% of B, for 4, and 23 min 75% of B.
c. Polar metabolites data analysis: the data processing was done using TraceFinder (Thermo Fisher Scientific), and when detected, compounds were identified by the accurate mass, retention time, isotope pattern, and fragments and verified using the in-house-generated mass spectral library.

## Supporting Information Available

Materials and Methods, ATP curve showing a linear increase in the luminescence signal with the increasing ATP concentration, ATP-synthesis activity of P-loop prototypes, global electrostatic surface potential of P-loop prototypes, ATP-synthesis activity of P-loop prototypes in the presence of inorganic polyphosphates, metal-dependent ATP-synthesis activity, temperature optimization of ATP-synthesis activity, inhibition of ATP-synthesis activity by AMP and APPcP, ATP-synthesis activity of the N-αβα prototype, time course analysis of the ATP-synthesis activity of the N-αβα prototype, mass distribution histograms of the N-αβα prototype using mass photometry analysis, ATP-synthesis activity of wild-type and mutant prototypes, ATP standard curves from a freshly prepared luciferase mix and after 3 h of incubation in the dark and on ice, purification of the N-αβα prototype by size exclusion chromatography, DNA sequences of P-loop prototypes, amino acid sequences of P-loop prototypes, and rates of ATP-synthesis activity of the N-half prototype (PDF)

## References

[ref1] Knowles J. R. Enzyme-Catalyzed Phosphoryl Transfer Reactions. Annu. Rev. Biochem. 1980, 49, 877–919. 10.1146/annurev.bi.49.070180.004305. 6250450

[ref2] Lassila J. K.; Zalatan J. G.; Herschlag D. Biological phosphoryl-transfer reactions: understanding mechanism and catalysis. Annu. Rev. Biochem. 2011, 80, 669–702. 10.1146/annurev-biochem-060409-092741. 21513457 PMC3418923

[ref3] Walker J. E.; Saraste M.; Runswick M. J.; Gay N. J. Distantly related sequences in the alpha- and beta-subunits of ATP synthase, myosin, kinases and other ATP-requiring enzymes and a common nucleotide binding fold. EMBO J. 1982, 1, 945–951. 10.1002/j.1460-2075.1982.tb01276.x. 6329717 PMC553140

[ref4] Leipe D. D.; Wolf Y. I.; Koonin E. V.; Aravind L. Classification and evolution of P-loop GTPases and related ATPases. J. Mol. Biol. 2002, 317, 41–72. 10.1006/jmbi.2001.5378. 11916378

[ref5] Leipe D. D.; Koonin E. V.; Aravind L. Evolution and classification of P-loop kinases and related proteins. J. Mol. Biol. 2003, 333, 781–815. 10.1016/j.jmb.2003.08.040. 14568537

[ref6] Ponting C. P.; Russell R. R. The Natural History of Protein Domains. Annu. Rev. Biophys. Biomol. Struct. 2002, 31, 45–71. 10.1146/annurev.biophys.31.082901.134314. 11988462

[ref7] Westheimer F. H. Why nature chose phosphates. Science 1987, 235, 1173–1178. 10.1126/science.2434996. 2434996

[ref8] Ogura T.; Wilkinson A. J. AAA+ superfamily ATPases: common structure–diverse function. Genes Cells 2001, 6, 575–597. 10.1046/j.1365-2443.2001.00447.x. 11473577

[ref9] Walker J. E. ATP Synthesis by Rotary Catalysis (Nobel lecture). Angew. Chem., Int. Ed. 1998, 37, 2308–2319. 10.1002/(sici)1521-3773(19980918)37:17<2308::aid-anie2308>3.0.co;2-w. 29710950

[ref10] Eck R. V.; Dayhoff M. O. Evolution of the structure of ferredoxin based on living relics of primitive amino Acid sequences. Science 1966, 152, 363–366. 10.1126/science.152.3720.363. 17775169

[ref11] Alva V.; Söding J.; Lupas A. N. A vocabulary of ancient peptides at the origin of folded proteins. eLife 2015, 4, e09410 10.7554/eLife.09410.001. 26653858 PMC4739770

[ref12] Frenkel-Pinter M.; Samanta M.; Ashkenasy G.; Leman L. J. Prebiotic Peptides: Molecular Hubs in the Origin of Life. Chem. Rev. 2020, 120, 4707–4765. 10.1021/acs.chemrev.9b00664. 32101414

[ref13] Tretyachenko V.; Vymětal J.; Bednárová L.; et al. Random protein sequences can form defined secondary structures and are well-tolerated in vivo. Sci. Rep. 2017, 7, 15449 10.1038/s41598-017-15635-8. 29133927 PMC5684393

[ref14] Saraste M.; Sibbald P. R.; Wittinghofer A. The P-loop - a common motif in ATP- and GTP-binding proteins. Trends Biochem. Sci. 1990, 15, 430–434. 10.1016/0968-0004(90)90281-F. 2126155

[ref15] Allen K. N.; Dunaway-Mariano D. Catalytic scaffolds for phosphoryl group transfer. Curr. Opin. Struct. Biol. 2016, 41, 172–179. 10.1016/j.sbi.2016.07.017. 27526404 PMC5154885

[ref16] Watson J. D.; Milner-White E. J. A novel main-chain anion-binding site in proteins: the nest. A particular combination of φ,ψ values in successive residues gives rise to anion-binding sites that occur commonly and are found often at functionally important regions 1 1Edited by J. Thornton. J. Mol. Biol. 2002, 315, 171–182. 10.1006/jmbi.2001.5227. 11779237

[ref17] Longo L. M.; Petrović D.; Kamerlin S. C. L.; Tawfik D. S. Short and simple sequences favored the emergence of N-helix phospho-ligand binding sites in the first enzymes. Proc. Natl. Acad. Sci. 2020, 117, 5310–5318. 10.1073/pnas.1911742117. 32079722 PMC7071883

[ref18] Ma B.; Chen L.; Ji H.; et al. Characters of very ancient proteins. Biochem. Biophys. Res. Commun. 2008, 366, 607–611. 10.1016/j.bbrc.2007.12.014. 18073136

[ref19] Koonin E. V. Comparative genomics, minimal gene-sets and the last universal common ancestor. Nat. Rev. Microbiol. 2003, 1, 127–136. 10.1038/nrmicro751. 15035042

[ref20] Söding J.; Lupas A. N. More than the sum of their parts: On the evolution of proteins from peptides. BioEssays 2003, 25, 837–846. 10.1002/bies.10321. 12938173

[ref21] Berezovsky I. N. Towards descriptor of elementary functions for protein design. Curr. Opin. Struct. Biol. 2019, 58, 159–165. 10.1016/j.sbi.2019.06.010. 31352188

[ref22] White H. B. Coenzymes as fossils of an earlier metabolic state. J. Mol. Evol. 1976, 7, 101–104. 10.1007/BF01732468. 1263263

[ref23] Romero Romero M. L.; Yang F.; Lin Y.-R.; et al. Simple yet functional phosphate-loop proteins. Proc. Natl. Acad. Sci. 2018, 115, E11943–E11950. 10.1073/pnas.1812400115. 30504143 PMC6304952

[ref24] Longo L. M.; Jabłońska J.; Vyas P.; On the Emergence of P-Loop NTPase and Rossmann Enzymes from a Beta-Alpha-Beta Ancestral Fragment; Deane C. M., Boudker O., Eds.; eLife, 2020; Vol. 9, p e64415. 10.7554/eLife.64415 PMC7758060 33295875

[ref25] Laurino P.; Tóth-Petróczy Á.; Meana-Pañeda R.; Lin W.; Truhlar D. G.; Tawfik D. S. An Ancient Fingerprint Indicates the Common Ancestry of Rossmann-Fold Enzymes Utilizing Different Ribose-Based Cofactors. PLoS Biol. 2016, 14, e1002396 10.1371/journal.pbio.1002396. 26938925 PMC4777477

[ref26] Vyas P.; Trofimyuk O.; Longo L. M.; Deshmukh F. K.; Sharon M.; Tawfik D. S. Helicase-Like Functions in Phosphate Loop Containing Beta-Alpha Polypeptides. Proc. Natl. Acad. Sci. U. S. A. 2021, 118, e2016131118 10.1073/pnas.2016131118. 33846247 PMC8072362

[ref27] Kornberg A. Inorganic polyphosphate: Toward making a forgotten polymer unforgettable. J. Bacteriol. 1995, 177, 491–496. 10.1128/jb.177.3.491-496.1995. 7836277 PMC176618

[ref28] Ahn K.; Kornberg A. Polyphosphate kinase from Escherichia coli. Purification and demonstration of a phosphoenzyme intermediate. J. Biol. Chem. 1990, 265, 11734–11739. 10.1016/s0021-9258(19)38459-5. 2164013

[ref29] Branchini B. R.; Southworth T. L. A Highly Sensitive Biosensor for ATP Using a Chimeric Firefly Luciferase. Methods Enzymol. 2017, 589, 351–364. 10.1016/bs.mie.2017.01.004. 28336069

[ref30] Matte A.; Tari L. W.; Delbaere L. T. J. How do kinases transfer phosphoryl groups?. Structure 1998, 6, 413–419. 10.1016/S0969-2126(98)00043-4. 9562560

[ref31] Adams J. A. Kinetic and Catalytic Mechanisms of Protein Kinases. Chem. Rev. 2001, 101, 2271–2290. 10.1021/cr000230w. 11749373

[ref32] Weinreb V.; Carter C. W. Mg2+-free Bacillus stearothermophilus tryptophanyl-tRNA synthetase retains a major fraction of the overall rate enhancement for tryptophan activation. J. Am. Chem. Soc. 2008, 130, 1488–1494. 10.1021/ja076557x. 18173270 PMC2826132

[ref33] Noda L.; Kuby S. A. Adenosine Triphosphate-Adenosine Monophosphate Transphosphorylase (Myokinase). J. Biol. Chem. 1957, 226, 551–558. 10.1016/s0021-9258(18)64853-7. 13428785

[ref34] Noda L. Adenylate Kinase. Group Transfer Part A: Nucleotidyl Transfer Nucleosidyl Transfer Acyl Transfer Phosphoryl Transfer; Boyer P. D., Ed.; Academic Press, 1973; Vol. Vol 8, pp 279–305.

[ref35] Zheng J.; Trafny E. A.; Knighton D. R.; et al. 2.2 Å refined crystal structure of the catalytic subunit of cAMP-dependent protein kinase complexed with MnATP and a peptide inhibitor. Acta Crystallogr., Sect. D: Biol. Crystallogr. 1993, 49, 362–365. 10.1107/S0907444993000423. 15299527

[ref36] Waas W. F.; Dalby K. N. Physiological Concentrations of Divalent Magnesium Ion Activate the Serine/Threonine Specific Protein Kinase ERK2. Biochemistry 2003, 42, 2960–2970. 10.1021/bi027171w. 12627962

[ref37] Cook P. F.; Neville M. E. J.; Vrana K. E.; Hartl F. T.; Roskoski R. J. Adenosine cyclic 3’,5’-monophosphate dependent protein kinase: kinetic mechanism for the bovine skeletal muscle catalytic subunit. Biochemistry 1982, 21, 5794–5799. 10.1021/bi00266a011. 6295440

[ref38] Saylor P.; Wang C.; Hirai T. J.; Adams J. A. A Second Magnesium Ion Is Critical for ATP Binding in the Kinase Domain of the Oncoprotein v-Fps. Biochemistry 1998, 37, 12624–12630. 10.1021/bi9812672. 9730835

[ref39] Bao Z. Q.; Jacobsen D. M.; Young M. A. Briefly bound to activate: transient binding of a second catalytic magnesium activates the structure and dynamics of CDK2 kinase for catalysis. Structure 2011, 19, 675–690. 10.1016/j.str.2011.02.016. 21565702 PMC3462661

[ref40] Jacobsen D. M.; Bao Z.-Q.; O’Brien P.; Brooks C. L. I. I. I.; Young M. A. Price To Be Paid for Two-Metal Catalysis: Magnesium Ions That Accelerate Chemistry Unavoidably Limit Product Release from a Protein Kinase. J. Am. Chem. Soc. 2012, 134, 15357–15370. 10.1021/ja304419t. 22891849 PMC3446636

[ref41] Recabarren R.; Zinovjev K.; Tuñón I.; Alzate-Morales J. How a Second Mg2+ Ion Affects the Phosphoryl-Transfer Mechanism in a Protein Kinase: A Computational Study. ACS Catal. 2021, 11, 169–183. 10.1021/acscatal.0c03304.

[ref42] Schulz G. E.; Schiltz E.; Tomasselli A. G.; et al. Structural relationships in the adenylate kinase family. Eur. J. Biochem. 1986, 161, 127–132. 10.1111/j.1432-1033.1986.tb10132.x. 3023080

[ref43] Pal P. K.; Ma Z.; Coleman P. S. The AMP-binding domain on adenylate kinase. Evidence for a conformational change during binary-to-ternary complex formation via photoaffinity labeling analyses. J. Biol. Chem. 1992, 267, 25003–25009. 10.1016/s0021-9258(19)73997-0. 1460003

[ref44] Berg O. G.; Gelb M. H.; Tsai M.-D.; Jain M. K. Interfacial Enzymology: The Secreted Phospholipase A2-Paradigm. Chem. Rev. 2001, 101, 2613–2654. 10.1021/cr990139w. 11749391

[ref45] Seeger F.; Quintyn R.; Tanimoto A.; et al. Interfacial residues promote an optimal alignment of the catalytic center in human soluble guanylate cyclase: heterodimerization is required but not sufficient for activity. Biochemistry 2014, 53, 2153–2165. 10.1021/bi500129k. 24669844 PMC3985721

[ref46] Ben-David M.; Sussman J. L.; Maxwell C. I.; Szeler K.; Kamerlin S. C. L.; Tawfik D. S. Catalytic Stimulation by Restrained Active-Site Floppiness-The Case of High Density Lipoprotein-Bound Serum Paraoxonase-1. J. Mol. Biol. 2015, 427, 1359–1374. 10.1016/j.jmb.2015.01.013. 25644661

[ref47] Bar-Even A.; Milo R.; Noor E.; Tawfik D. S. The Moderately Efficient Enzyme: Futile Encounters and Enzyme Floppiness. Biochemistry 2015, 54, 4969–4977. 10.1021/acs.biochem.5b00621. 26219075

[ref48] Vidossich P.; Castañeda Moreno L. E.; Mota C.; de Sanctis D.; Miscione M. G.; De Vivo M. Functional Implications of Second-Shell Basic Residues for dUTPase DR2231 Enzymatic Specificity. ACS Catal. 2020, 10, 13825–13833. 10.1021/acscatal.0c04148.

[ref49] Debler E. W.; Müller R.; Hilvert D.; Wilson I. A. Conformational Isomerism Can Limit Antibody Catalysis. J. Biol. Chem. 2008, 283, 16554–16560. 10.1074/jbc.M710256200. 18417480 PMC2423248

[ref50] Sonn-Segev A.; Belacic K.; Bodrug T.; et al. Quantifying the heterogeneity of macromolecular machines by mass photometry. Nat. Commun. 2020, 11, 1772 10.1038/s41467-020-15642-w. 32286308 PMC7156492

[ref51] Ben-David M.; Elias M.; Filippi J.-J.; et al. Catalytic versatility and backups in enzyme active sites: the case of serum paraoxonase 1. J. Mol. Biol. 2012, 418, 181–196. 10.1016/j.jmb.2012.02.042. 22387469

[ref52] Jensen R. A. Enzyme Recruitment in Evolution of New Function. Annu. Rev. Microbiol. 1976, 30, 409–425. 10.1146/annurev.mi.30.100176.002205. 791073

[ref53] Deyrup A. T.; Krishnan S.; Cockburn B. N.; Schwartz N. B. Deletion and Site-directed Mutagenesis of the ATP-binding Motif (P-loop) in the Bifunctional Murine Atp-Sulfurylase/Adenosine 5′-Phosphosulfate Kinase Enzyme. J. Biol. Chem. 1998, 273, 9450–9456. 10.1074/jbc.273.16.9450. 9545271

[ref54] Chuang W. J.; Abeygunawardana C.; Gittis A. G.; Pedersen P. L.; Mildvan A. S. Solution Structure and Function in Trifluoroethanol of PP-50, an ATP-Binding Peptide from F1ATPase. Arch. Biochem. Biophys. 1995, 319, 110–122. 10.1006/abbi.1995.1272. 7771774

[ref55] Pham Y.; Kuhlman B.; Butterfoss G. L.; Hu H.; Weinreb V.; Carter C. W. J. Tryptophanyl-tRNA Synthetase Urzyme. J. Biol. Chem. 2010, 285, 38590–38601. 10.1074/jbc.M110.136911. 20864539 PMC2992291

[ref56] Castillo-Caceres C.; Duran-Meza E.; Nova E.; Araya-Secchi R.; Monasterio O.; Diaz-Espinoza R. Functional characterization of the ATPase-like activity displayed by a catalytic amyloid. Biochim. Biophys. Acta, Gen. Subj. 2021, 1865, 129729 10.1016/j.bbagen.2020.129729. 32916204

[ref57] Richard J. P. Enabling Role of Ligand-Driven Conformational Changes in Enzyme Evolution. Biochemistry 2022, 61, 1533–1542. 10.1021/acs.biochem.2c00178. 35829700 PMC9354746

[ref58] Orgel Leslie E. Prebiotic Chemistry and the Origin of the RNA World. Crit. Rev. Biochem. Mol. Biol. 2004, 39, 99–123. 10.1080/10409230490460765. 15217990

[ref59] Stairs S.; Nikmal A.; Bučar D.-K.; Zheng S.-L.; Szostak J. W.; Powner M. W. Divergent prebiotic synthesis of pyrimidine and 8-oxo-purine ribonucleotides. Nat. Commun. 2017, 8, 15270 10.1038/ncomms15270. 28524845 PMC5454461

[ref60] Lin H.; Jiménez E. I.; Arriola J. T.; Müller U. F.; Krishnamurthy R. Concurrent Prebiotic Formation of Nucleoside-Amidophosphates and Nucleoside-Triphosphates Potentiates Transition from Abiotic to Biotic Polymerization. Angew. Chem., Int. Ed. 2022, 61, e202113625 10.1002/anie.202113625. 34738300

[ref61] Atkinson D. E.; Walton G. M. Adenosine Triphosphate Conservation in Metabolic Regulation. J. Biol. Chem. 1967, 242, 3239–3241. 10.1016/s0021-9258(18)95956-9. 6027798

[ref62] Albery W. J.; Knowles J. R. Evolution of Enzyme Function and the Development of Catalytic Efficiency. Biochemistry 1976, 15, 5631–5640. 10.1021/bi00670a032. 999839

[ref63] Noda-Garcia L.; Liebermeister W.; Tawfik D. S. Metabolite-Enzyme Coevolution: From Single Enzymes to Metabolic Pathways and Networks. Annu. Rev. Biochem. 2018, 87, 187–216. 10.1146/annurev-biochem-062917-012023. 29925259

[ref64] Freire M. Á. Short non-coded peptides interacting with cofactors facilitated the integration of early chemical networks. Biosystems 2022, 211, 104547 10.1016/j.biosystems.2021.104547. 34547425

[ref65] Nobeli I.; Ponstingl H.; Krissinel E. B.; Thornton J. M. A structure-based anatomy of the E.coli metabolome. J. Mol. Biol. 2003, 334, 697–719. 10.1016/j.jmb.2003.10.008. 14636597

[ref66] Hirsch A. K. H.; Fischer F. R.; Diederich F. Phosphate recognition in structural biology. Angew. Chem., Int. Ed. Engl. 2007, 46, 338–352. 10.1002/anie.200603420. 17154432

[ref67] Noor E.; Flamholz A. I.; Jayaraman V.; et al. Uniform binding and negative catalysis at the origin of enzymes. Protein Sci. 2022, 31, e4381 10.1002/pro.4381. 35900021 PMC9281367

[ref68] Whitford P. C.; Gosavi S.; Onuchic J. N. Conformational Transitions in Adenylate Kinase. J. Biol. Chem. 2008, 283, 2042–2048. 10.1074/jbc.M707632200. 17998210

[ref69] Mulkidjanian A. Y.; Makarova K. S.; Galperin M. Y.; Koonin E. V. Inventing the dynamo machine: the evolution of the F-type and V-type ATPases. Nat. Rev. Microbiol. 2007, 5, 892–899. 10.1038/nrmicro1767. 17938630

[ref70] Fontecilla-Camps J. C. Primordial bioenergy sources: The two facets of adenosine triphosphate. J. Inorg. Biochem. 2021, 216, 111347 10.1016/j.jinorgbio.2020.111347. 33450675

[ref71] Zheng L.; Cardaci S.; Jerby L.; et al. Fumarate induces redox-dependent senescence by modifying glutathione metabolism. Nat. Commun. 2015, 6, 6001 10.1038/ncomms7001. 25613188 PMC4340546

