# Supplementary material for: On the Origins of Enzymes: Phosphate-Binding Polypeptides Mediate Phosphoryl Transfer to Synthesize Adenosine Triphosphate
Source: J Am Chem Soc. 2023 Mar 23;145(15):8344–54. doi: 10.1021/jacs.2c08636 (PMC10119929; doi:10.1021/jacs.2c08636)
Supplement: Supplementary file 1 — ja2c08636_si_001.pdf [file ja2c08636_si_001.pdf]

# Supplementary Information
# For
# ‘On the Origins of Enzymes: Phosphate-binding Polypeptides Mediate Phosphoryl Transfer to Synthesize Adenosine Triphosphate’

Pratik Vyas*, Sergey Malitsky, Maxim Itkin and Dan S. Tawfik

*Corresponding author. Email: pratik.vyas@weizmann.ac.il; prateekvyas07@gmail.com

This PDF file includes:

I. Materials and Methods
II. Figures S1 to S13
III. Tables S1 to S3
IV. References for supplementary information

# I. SUPPLEMENTARY MATERIALS AND METHODS

**Materials**: Recombinant luciferase from Photinus pyralis firefly (Sigma SRE0045) was dissolved in 1 M Tris (pH 7.5) to prepare 1 mg/ml stock solution. D-luciferin, potassium salt (*GoldBio* LUCK-100) was dissolved in 50 mM Tricine (pH 7.6) to prepare 10 mM stock solution. Adenosine triphosphate (Sigma, > 95% pure), adenosine diphosphate (*EMD Millipore-Merck,* 98% pure), adenosine monophosphate (Sigma, > 95% pure), sodium triphosphate pentabasic (Sigma, ≥ 98% pure) and sodium polyphosphate (EMPLURA® - Graham's salt; ~25 mer) were each dissolved in 50 mM Tricine buffer (pH 7.6) to prepare stock solutions at 50 mM stock concentration. Stocks were divided into aliquots, flash frozen and stored at -20 °C (luciferase, ATP, ADP and polyphosphate) and at -80 °C (luciferin). Fresh aliquots were used for each experiment.

**Methods: Optimization of reaction conditions for ATP synthesis** (ADP to ATP *plus* AMP conversion):

*i. Metal dependence (Supplementary Figure S5):*

The N-half prototype was incubated with presumed saturating concentration of ADP (1 mM) with varying concentrations of metal salts (0-20 mM) in 50 mM tricine buffer (pH 7.6) in 100 μl of reaction volumes at 37°C for 1 hour. 3 μl of test reaction was transferred to 384-well white plate to which 30 μl of luciferase premix (3.2 μM luciferase, 370 μM luciferin, 10 mM $MgCl_2$ in 50 mM Tricine, pH 7.6) was added. Luminescence was measured in *Tecan Infinite M-Plex* plate reader with 'automatic attenuation' setting and 100 millisecond integration time. In parallel, luminescence from ATP dilutions (with

1 mM ADP with respective metal salt) was measured to generate a standard curve that was used to quantify the ATP produced in test reactions.

*ii. Determination of fraction of free and magnesium bound ADP (Supplementary Figure S5A):*

We calculated the concentrations of free ADP, Mg.ADP and free $MgCl_2$ using the standard formula for Kd:

$$KD_{Mg.ADP} = \frac{[Mg^{+2}][ADP]}{[Mg.ADP]} \quad \text{Eq. 1}$$

Rewriting equation 1:

$$KD_{Mg.ADP} = \frac{[Mg^{+2} - x][ADP - x]}{[x]} \quad \text{Eq. 2}$$

where $[x]$ is the concentration of Mg.ADP, $Mg^{+2}$ is the concentration of $MgCl_2$ (0-20000 μM) and ADP takes a constant value of 1000 μM (i.e., the ADP concentration in the reaction mixture).

Rearranging equation 2,

$$[KD_{Mg.ADP}][x] = [Mg^{+2} - x][ADP - x] \quad \text{Eq. 3}$$

The dissociation constants for Mg.ADP reported in the literature ranged from ~200 to 900 μM: Gupta and Benkovic [1] – 430 ± 30 μM; J. Blair [2] (and the references therein) - 233 μM, 250 μM, 289 μM and 316 μM; Chinopoulos et.al.[3] - 906 ± 23 μM**;** Gout et. al.[4] - 670 ± 50 μM**;** K. Burton [5] - 450 μM**.** Therefore, we assumed an average value of 443 μM.

Therefore, rewriting equation 3:

$$443\,x = [Mg^{+2} - x][ADP - x] \quad \text{Eq. 4}$$

Equation 4 is a quadratic equation that can be solved for $[x]$ by plugging in the known concentration values of ADP and $MgCl_2$. The calculated values of Mg.ADP in the reaction pool, at a given concentration of $MgCl_2$, are described in the table below. Free ADP was calculated by subtracting the value of Mg.ADP from the total ADP concentration (1000 μM), whereas free $MgCl_2$ in the reaction was calculated by subtracting the value of Mg.ADP from the concentration of $MgCl_2$ (assuming 1:1 stoichiometry of Mg:ADP). The values of initial velocity, Mg.ADP, free ADP and free $MgCl_2$ shown in the table below are averaged values from two independent measurements (Supplementary Figure S5A)

| $MgCl_2$ (μM) (Supplementary Figure S5A) | Initial velocity (μM ATP.$s^{-1}$) | Mg.ADP (μM) | Free ADP (μM) $(1000 - \mathrm{Mg.ADP})$ | Free $MgCl_2$ (μM) (Assuming 1:1 Mg:ADP stochiometry | Mg.ADP X ADP $(\mu M)^2$ |
|---|---|---|---|---|---|
| 0 | 0.000403 | 0 | 1000 | 0 | 0 |
| 50 | 0.00302 | 34 | 966 | 16 | 32844 |
| 100 | 0.00425 | 65 | 935 | 35 | 60775 |
| 500 | 0.006278 | 305 | 695 | 195 | 211975 |
| 1000 | 0.006472 | 520 | 480 | 480 | 249600 |
| 2500 | 0.006139 | 793 | 207 | 1707 | 164151 |
| 5000 | 0.005056 | 902 | 98 | 4098 | 88396 |
| 10000 | 0.004972 | 953 | 47 | 9047 | 44791 |
| 15000 | 0.004556 | 969 | 31 | 14031 | 30039 |
| 20000 | 0.004472 | 977 | 23 | 19023 | 22471 |

The activity plateaus at 0.5 mM $MgCl_2$ and remains constant until 2.5 mM $MgCl_2$ as the product of the two substrates (last column) remains roughly constant. In the regime from 5 to 20 mM $MgCl_2$, Mg.ADP increases and ADP depletes from the reaction. This is concomitant with an increase in free $MgCl_2$ in the reaction. The presence of excess free $MgCl_2$ likely allows the prototypes to accommodate an addition $Mg^{+2}$ ion in the active site. As described in the main text the presence of additional $Mg^{+2}$ ion in the active site

can improve catalytic functions and therefore compensate for a possible decrease in activity due to depleted ADP levels.

*iii.* Temperature and pH optimization:

For temperature optimization (**Supplementary Figure S6A**), 4 μM of N-half prototype was incubated with 1 mM ADP and 0.5 mM $MgCl_2$ (as optimized from the above experiment) in 50 mM tricine buffer (pH 7.6) at varying temperatures. 30 μl of test reactions were transferred to 96-well white plate and luminescence was measured, using *Tecan Infinite M-Plex* plate reader as above, at varying time points (5, 30 and 60 min). ATP generated in the reaction was quantified by an ATP standard curve (as described above; **Supplementary Figure S1A**)

For pH calibration (**Supplementary Figure S6B**), 4 μM of N-half prototype was incubated with 1 mM ADP, 0.5 mM $MgCl_2$ at 37°C for 1 hr. in 50 mM tricine buffer with varying pH (6.6 to 8.8). For lower pH conditions (pH 4.5, 5 and 6), proteins were incubated in 50 mM MES buffer having the corresponding pH. After the 1 hr. incubation, 3 μl of test reactions were transferred to 384-well white plates and luminescence was measured as described above by adding 30 μl luciferase premix. To quantify ATP generated in the reaction for each pH condition, ATP dilutions (for standard curve) were also made in the buffer with corresponding pH.

**Mass-photometry (Supplementary Figure S10):**

Mass photometry data were collected using One$^{MP}$ mass photometer (Refeyn Ltd) using previously published protocols [6]. In brief, gaskets (with chambers for holding the protein solution) were washed, dried and assembled on a clean glass overslip. Next, 16 μl of 50

mM tricine buffer (filtered through 3K MWCO centrifugal filters) was loaded in one of the chambers to find camera focus. For mass calibration, 4 μl of 50 nM urease standard was added to the same chamber and the video was recorded for a period of 120 s. Next, in a clean chamber on the gasket, 16 μl of 50 mM tricine buffer was added and camera was adjusted to find the right focus. Then, N-αβα prototype was mixed with 1 mM ADP and 0.5 mM $MgCl_2$ and 4 μl aliquot (200 nM final concentration) was immediately transferred to the same chamber. Video recorded for a period of 120 s. The same process was performed after incubating the reaction mix at 37 °C for 120 mins. Control measurement (N-αβα incubated at 37 °C for 120 min.) was performed as described above. The videos were processed and analyzed using Discover$^{MP}$ software, wherein histograms based on the observed masses, the bin width and the window range are built and fit to a Gaussian function.

**Size-exclusion chromatography (Supplementary Figure S13):** To test if the specific activity improves upon further round of purification, N-αβα prototype was purified via guanidine hydrochloride refolding/NiNTA method (see main text 'Methods'). Peak elution fractions were subjected to two rounds of dialysis in 50 mM Tris, 100 mM NaCl (pH 8) buffer. The protein precipitated during dialysis and was collected by centrifugation. The protein pellet was re-solubilized in 50 mM Tris, 100 mM NaCl, 1 M L-arginine (pH 7) buffer. Superdex 75 Increase 10/300 column (cytiva™) was equilibrated with 1.5 column volumes with the same buffer (50 mM Tris, 100 mM NaCl, 1 M L-arginine (pH 7)) using ÄKTA™ pure chromatography system. 200 μl of purified N-αβα prototype (600 μM) was injected on to the column and 500 μl elution fractions were collected. The protein eluted predominantly around the 10 ml mark (~ 75 kDa) that is in-

line with previous mass spectrometry results in that N-αβα prototype can form 10-mer assemblies (~ 67 kDa) [7]. L-arginine in the peak fraction was dialyzed out by two rounds of dialysis: 3 hours at room temperature followed by overnight dialysis, at 4 °C, in 50 mM Tricine buffer (pH 7.6). The dialyzed fraction was tested for activity by standard procedure as described earlier.

## II. SUPPLEMENTARY FIGURES

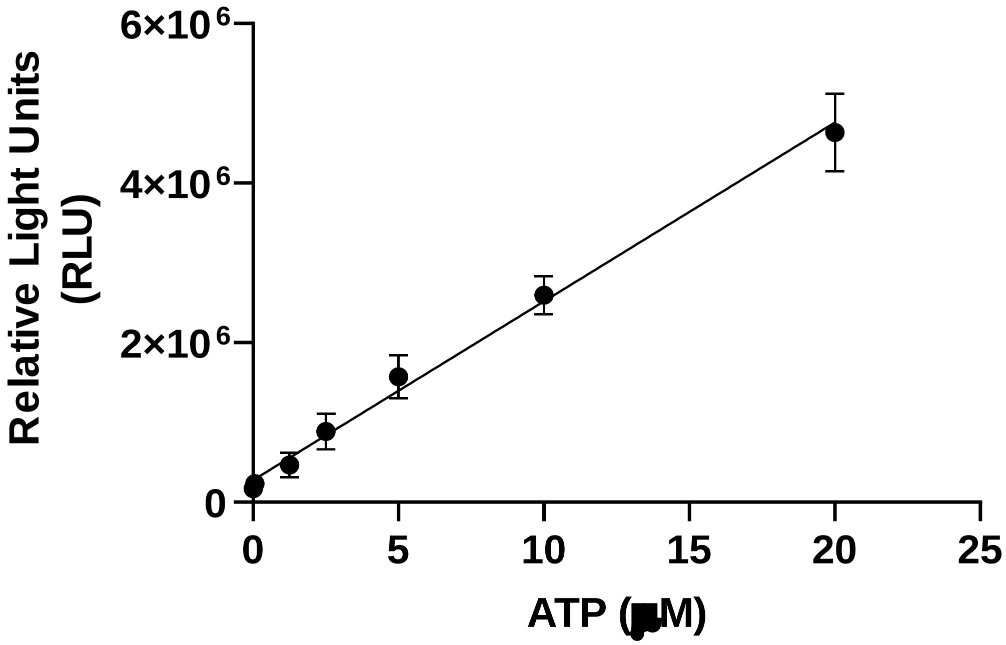


**Figure S1: A representative standard curve.** ATP curve showing linear increase in luminescence signal (relative light units/RLU) with increasing ATP concentration. ATP curve was generated by measuring luminescence signal upon addition of 30 µl of luciferase reaction mix (3.2 µM luciferase, 370 µM luciferin, 10 mM $MgCl_2$) to 30 µl of ATP dilutions containing 1 mM ADP and 0.5 mM $MgCl_2$. Error bars represent standard deviation from three to six independent measurements. $R^2$ = 0.9715; $p < 0.0001$ (calculated using GraphPad Prism software (8.3.0)).

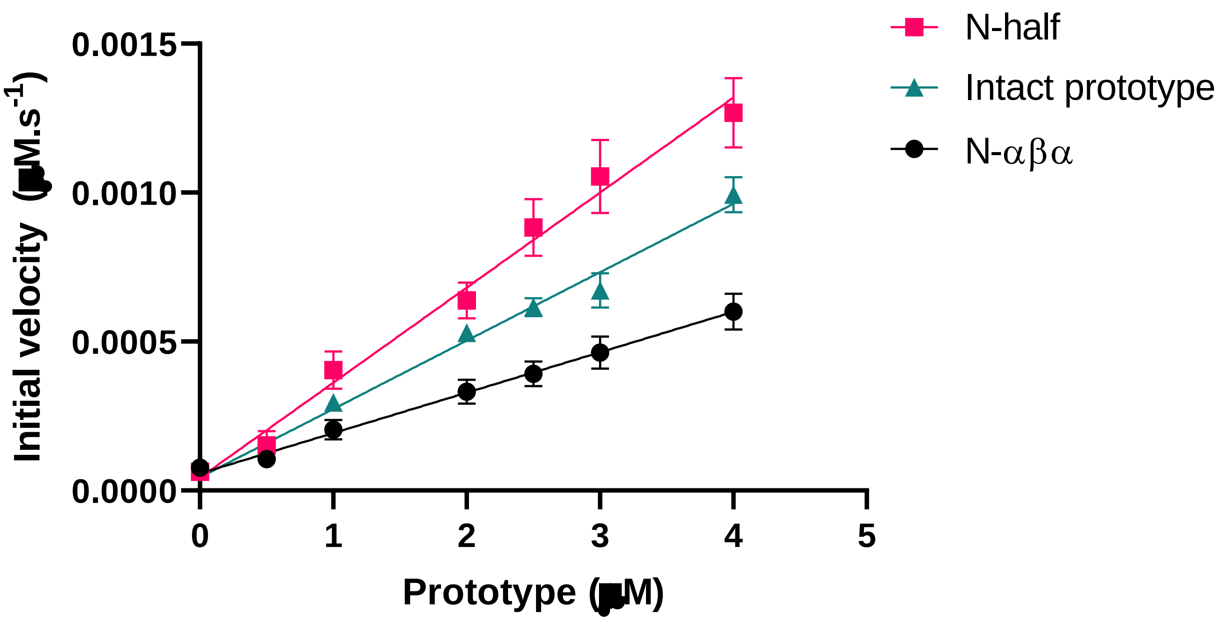


**Figure S2: The ATP-synthesis activity of P-loop prototypes*.** Luciferase assay showing linear increase in initial velocity (µM ATP synthesized per second) with increasing concentration of prototypes. N-half (pink squares); Intact prototype (turquoise triangles) and N-αβα (black circles). Reactions were carried out in presence of 1 mM ADP and 0.5 mM $MgCl_2$ in tricine buffer (pH 7.6). Reactions were incubated at 37 °C for 1 hr. (see 'Methods'). Error bars represent standard error of mean (SEM) from four to eight independent experiments.

***P-loop prototypes, especially the short prototypes, are likely to exist and various conformational and oligomeric states. Therefore, a shorter construct may adopt a conformational state that is more favorable for phosphoryl-transfer activity relative to the intact constructs. This may explain why the larger, intact, prototype appears to have slightly weak ATP-synthesis activity than the shorter N-half construct.

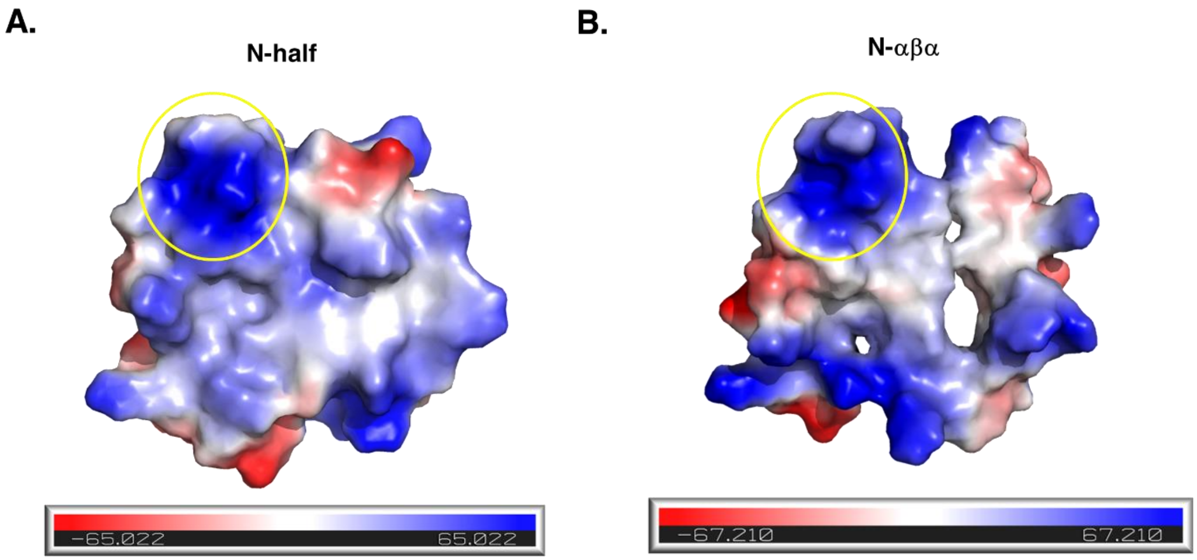


**Figure S3: The global electrostatic surface potential of P-loop prototypes.** Electrostatic surface potential was calculated using PyMol (pymol.org). Blue surfaces indicate positive charge and red surfaces indicate negative charge. The P-loop region, circled yellow, indicates high positive charge in both N-half and N-αβα prototypes.

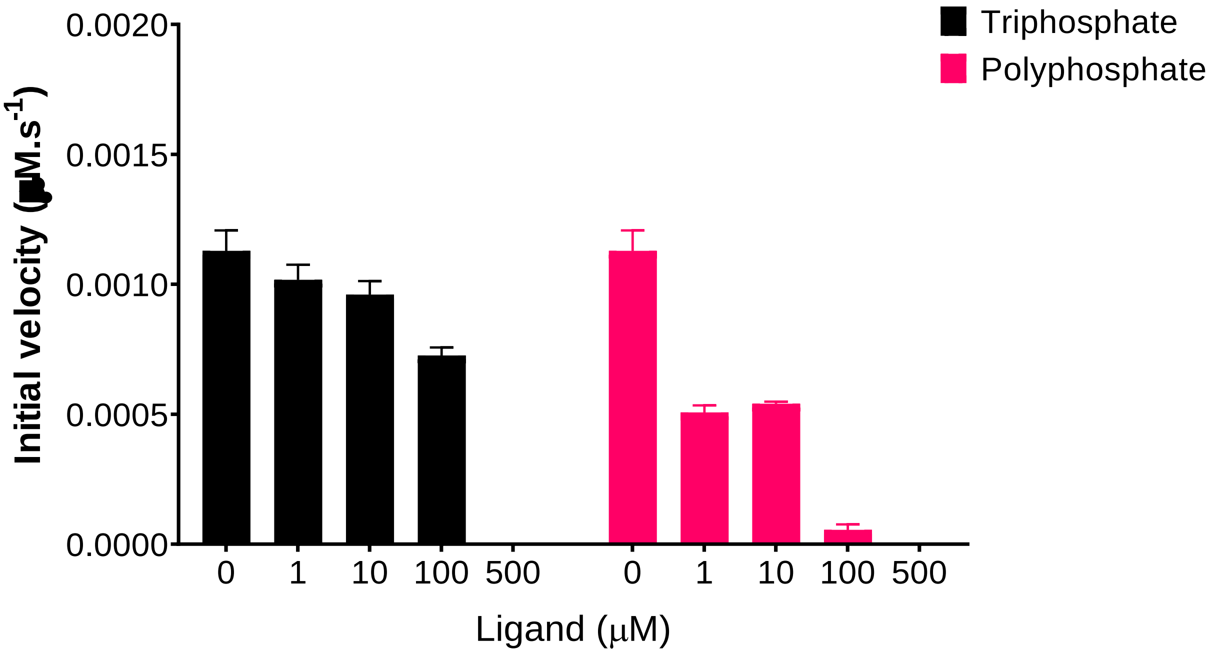


**Figure S4: The ATP-synthesis activity of P-loop prototypes in presence of inorganic polyphosphates.** The N-αβα prototype (4 μM) was incubated with 1 mM ADP and 0.5 mM $MgCl_2$ with varying concentrations (1-500 μM) of inorganic triphosphates (black bars) and polyphosphates (25-mer) (pink bars). Reactions were carried out in 50 mM tricine buffer (pH 7.6). Shown here are initial velocities of ATP synthesis ($\mu M.s^{-1}$) plotted against ligand concentration (μM).

**A.**

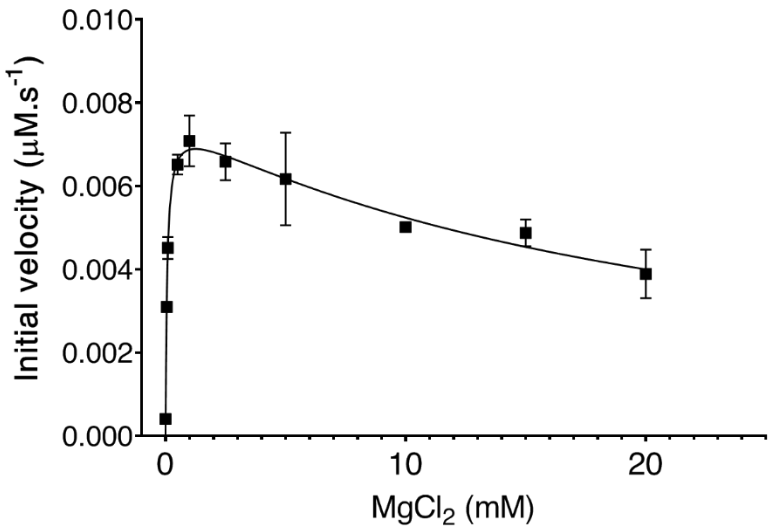


**B.**

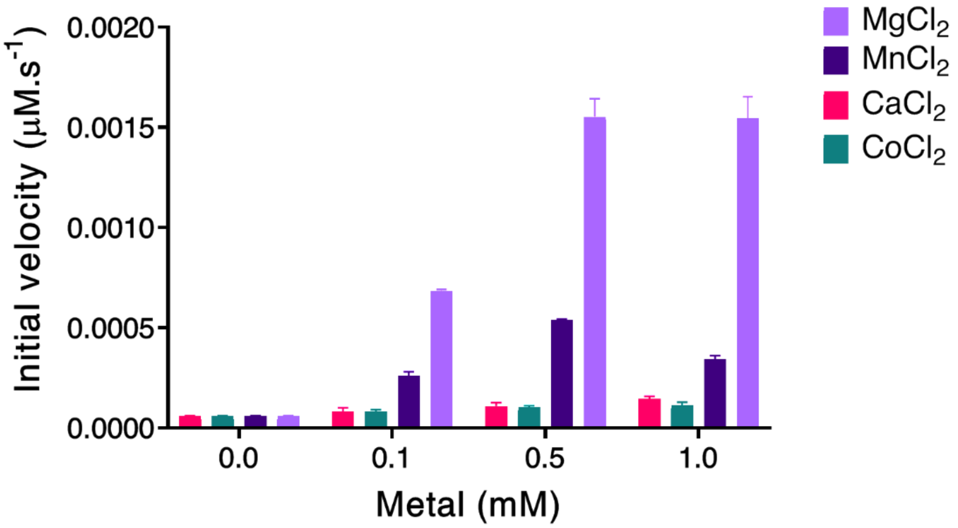


**Figure S5: The metal-dependent ATP-synthesis activity. A.** Determining optimal magnesium concentration for ATP-synthesis activity (expressed as $\mu M.s^{-1}$) of N-half prototype (10 μM). Shown here are the end-point values (after 1 hr. incubation) of ATP produced in the reaction (black bars). Data were fit to standard substrate inhibition equation using GraphPad Prism (8.3.0) to determine the apparent Km ($Km_{app}$) of $MgCl_2$. The calculated $Km_{app}$ for $MgCl_2$ was 70 (10) μM; the value in parenthesis represents standard deviation from two independent measurements. **B.** ATP-synthesis activity ($\mu M.s^{-1}$) of N-half prototype (4 μM) in presence of

different metal salts at 0, 0.1, 0.5 and 1 mM concentration. In both A and B, N-half prototype was incubated with a constant concentration of ADP (1 mM) and titrated with varying concentrations of metal salts. The reactions were incubated at 37°C for 1 hr. and the ATP generated in the reaction was calculated by a standard ATP curve (Supplementary Figure S1). Error bars represent standard deviation from two independent experiments. In addition to the metal salts depicted in the figure, we also tested $FeCl_3$, $NiCl_2$, $SrCl_2$ and $CuCl_2$, for which we did not observe any activity.

A.

B.

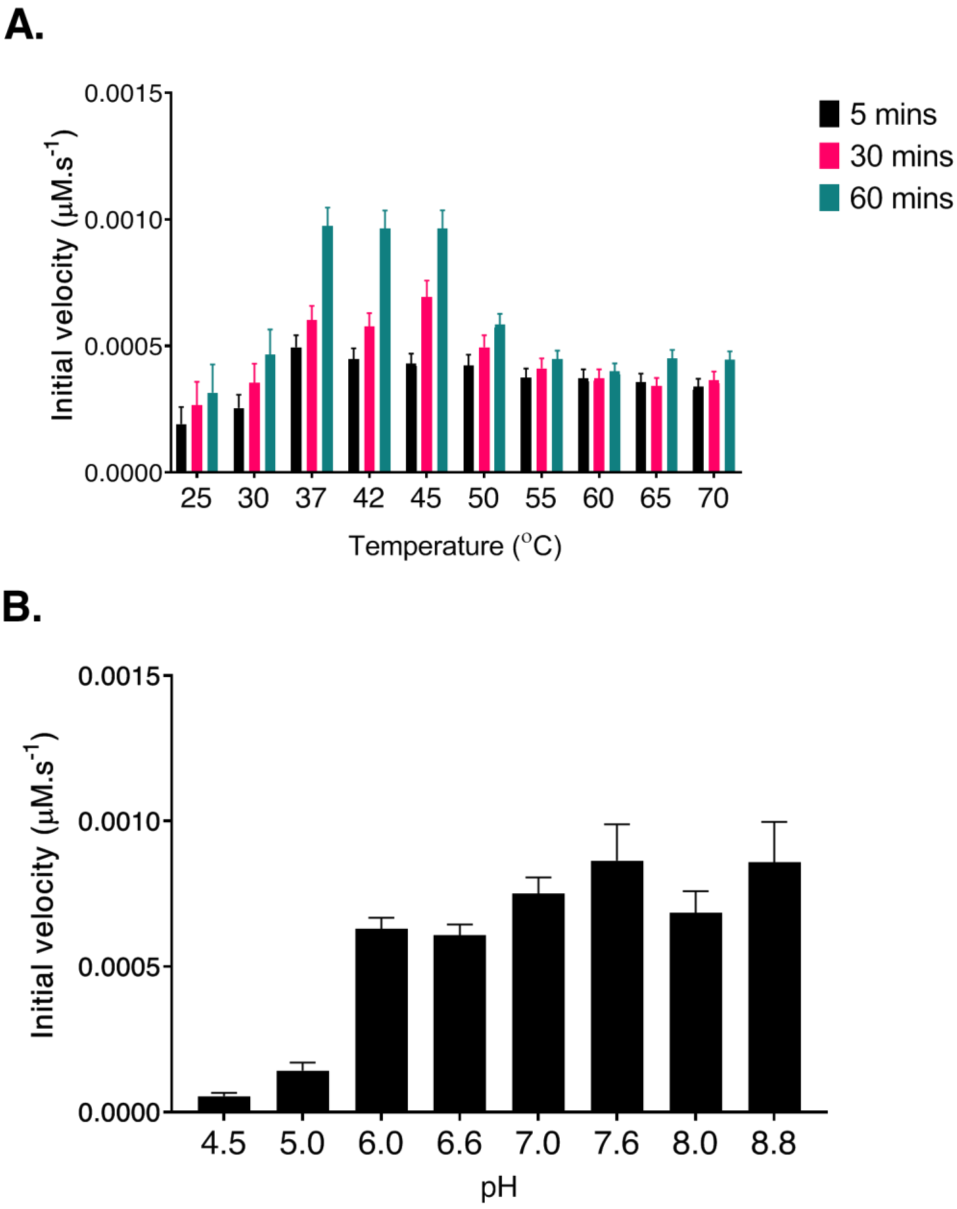


**Supplementary Figure S6: A. Temperature optimization of ATP-synthesis activity (expressed as μM.s$^{-1}$).** The N-half prototype (4 μM) was incubated with 1 mM ADP and 0.5 mM $MgCl_2$ in 50 mM tricine buffer (pH 7.6) at varying temperatures. Luminescence was measured at 5 mins (black bars), 30 mins (pink bars) and 60 mins (turquoise bars). ATP produced in the reaction was quantified as described in supplementary methods section. **B. pH calibration of ATP-synthesis activity (expressed as μM.s$^{-1}$).** N-half prototype (4 μM) was incubated with 1 mM ADP and 0.5 mM $MgCl_2$ at 37°C in buffers with varying pH for 1 hr. For pH range 6.6 to 8.8, 50 mM tricine buffer was used, whereas lower pH reactions (4.5, 5 and 6) were performed in 50 mM MES buffer. ATP produced in the reactions, in A and B, was quantified by a standard curve

(as described in Supplementary Methods section). Error bars in A and B represent standard deviation from two independent experiments.

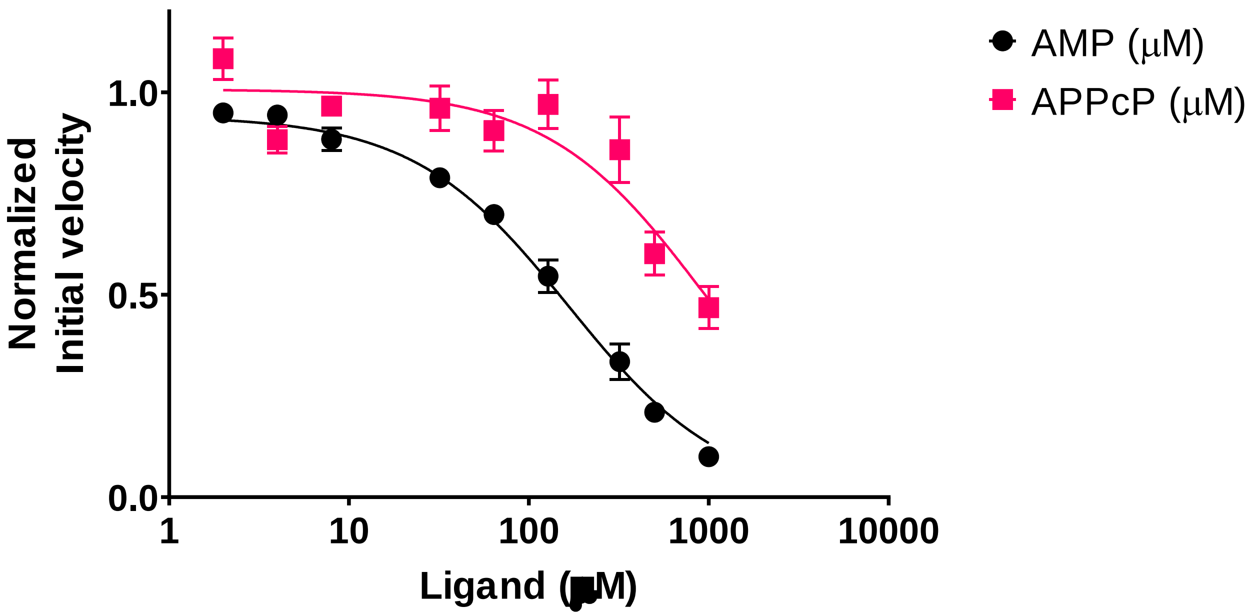


**Figure S7: Inhibition of the ATP-synthesis activity by AMP and APPcP.** The N-αβα prototype (5 μM) was incubated with 1 mM ADP, 0.5 mM $MgCl_2$ and varying concentrations of APPcP (non-hydrolysable analog of ATP) or AMP in 50 mM tricine buffer (pH 7.6) at 37°C for 1 hr. ATP synthesized in the reaction was measured using luciferase assay and quantified using ATP standard curves as described earlier. A standard curve was generated for each ligand concentration (AMP and APPcP) to correct for variabilities in luciferase activity due to AMP or APPcP. Initial velocities were normalized to the reaction without AMP or APPcP. Normalized velocities were then plotted as function of ligand (AMP or APPcP) concentration. Data were fit to standard 'Morrison equation for tight binding' using GraphPad Prism (8.3.0) to determine inhibitor constants ($Ki_{app}$) for AMP and APPcP. Enzyme concentration was set to 5 μM (concentration of prototype), substrate concentration (ADP concentration) was set to 1000 μM and Km was set to 67 μM (ADP Km; Table 1; main text). The calculated $Ki_{app}$ values for AMP was 10 (1) μM and APPcP was 80 (20) μM. Error bars and values in parenthesis represent standard deviation from three independent measurements.

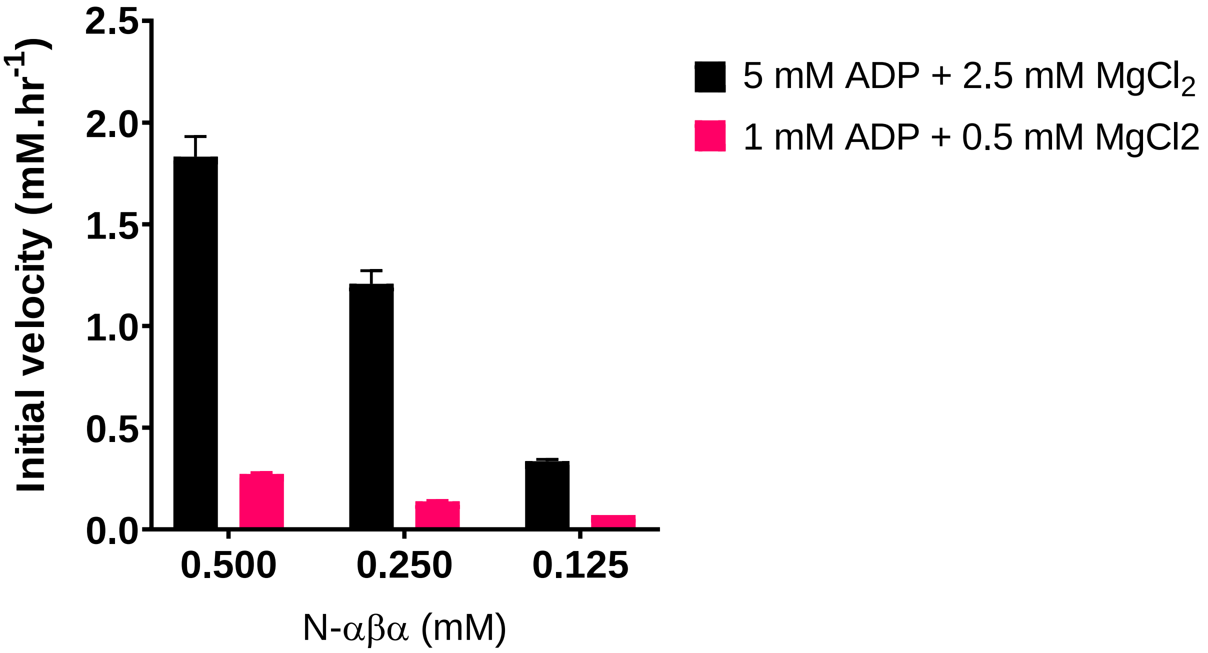


**Figure S8: The ATP-synthesis activity of N-αβα prototype**. To demonstrate conversion of larger fraction of ADP to ATP and faster initial rates, higher starting concentrations of N-αβα prototype were used (0.125, 0.250 and 0.500 mM). The prototypes generally precipitated during the dialysis step following purification and therefore, storage at high stock concentrations (i.e., 0.2 – 0.4 mM) required 1 M L-arginine to solubilize the precipitates. However, high levels of arginine salt (50 mM and above) interfered with experiments. Hence to circumvent the interference from arginine, supernatant (typically containing 0.05 to 0.1 mM of soluble protein) from the precipitated N-αβα proteins was collected and concentrated to 1.2 mM (in 50 mM tris buffer, pH 8) using 3 KDa cut-off centrifugal filters. Increasing concentrations of the N-αβα prototype (0.125, 0.250 and 0.500 mM) was incubated with 5 mM ADP and 2.5 mM $MgCl_2$ (black bars) or 1 mM ADP and 0.5 mM $MgCl_2$ (pink bars) at 37°C for 1 hr. in 50 mM tricine buffer (pH 7.6). ATP produced in the reaction was quantified as described earlier. Shown here is the initial velocity of ATP synthesis (expressed as $mM.h^{-1}$) plotted against N-αβα concentration. Error bars represent standard deviation from three to four independent experiments.

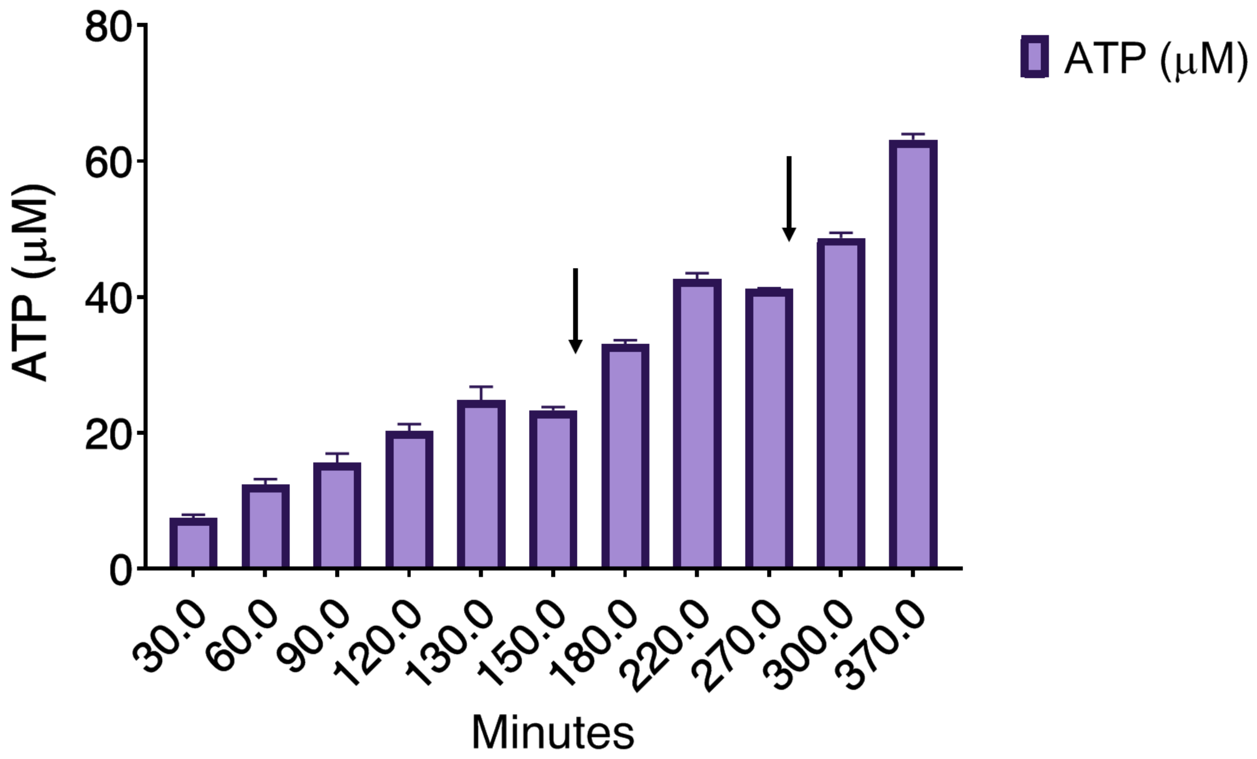


**Figure S9: A time-course analysis of the ATP-synthesis activity of N-αβα prototype**. N-αβα prototype (10 μM) was incubated with 1 mM ADP and 0.5 mM $MgCl_2$ at 37°C in 50 mM tricine buffer (pH 7.6). Luminescence was measured at varying time-points. ATP synthesized in the reaction was calculated using ATP standard curve (see 'Methods'). Each time the reaction reached a plateau, an aliquot of 10 μM prototype was added to the reaction (indicated by black arrows) and luminescence was further monitored. The reaction was monitored for 370 minutes.

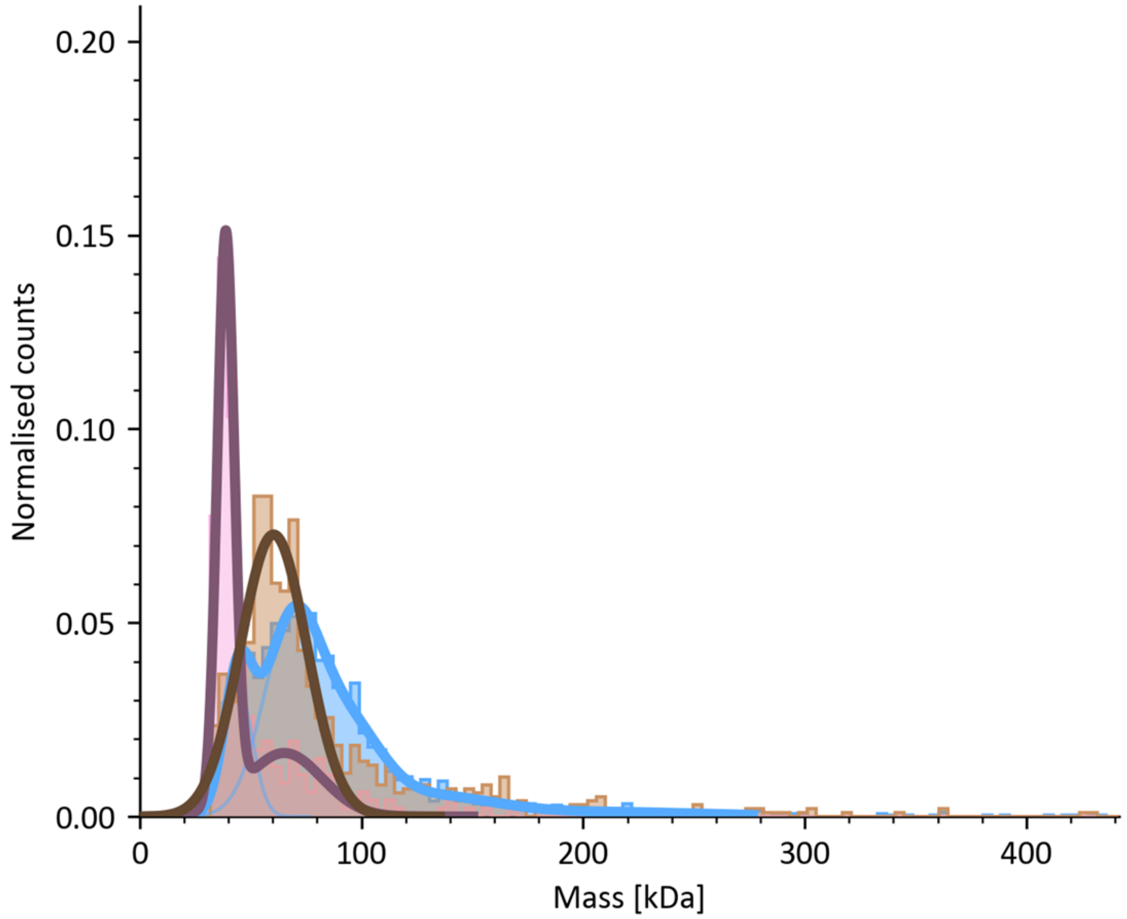


**Figure S10: Mass distribution histograms of N-αβα prototype using mass-photometry (MP) analysis[6].** Shown here are the most abundant mass (oligomer) species of N-αβα prototype immediately after mixing with ADP and $MgCl_2$ (peak at 60 kDa; brown gaussian fit) and after two-hour incubation at 37 ºC (Major peak at 39 kDa and minor peak at 60 kDa; purple gaussian fit). Blue gaussian fit corresponds to N-αβα prototype incubated, without ADP and MgCl2, at 37 ºC for two hours (peak at 65 kDa). Y-axis represent the number of measurements (counts) that fall in to a particular mass-range. Counts are normalised using Discover$^{MP}$ software. Normalisation divides the counts in each bin by the total number of events. This facilitates easier comparison between histograms with differing total numbers of events that can occur due to pipetting variabilities.

**A.**

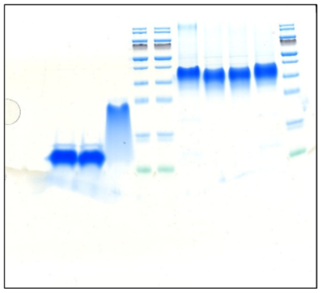

**B.**

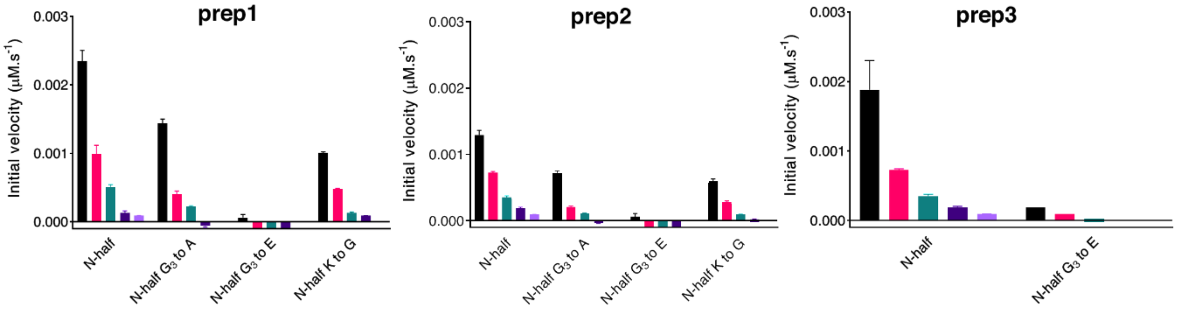


**C.**

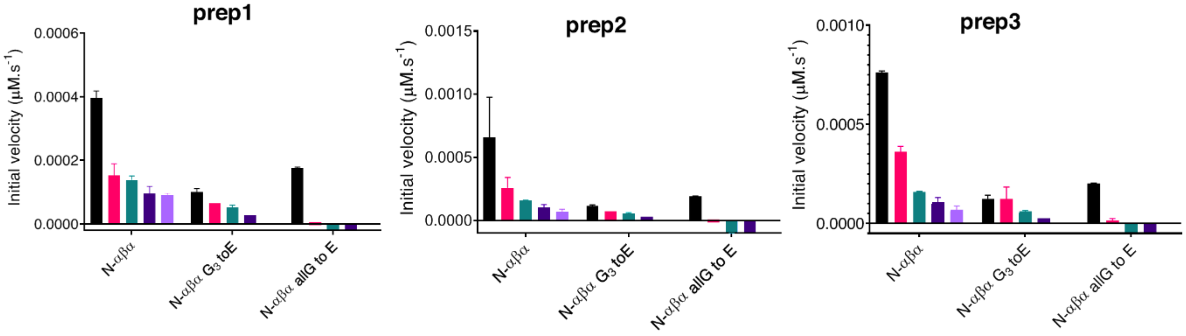


**Figure S11: The ATP-synthesis activity of wild type and mutant prototypes. A.** Representative SDS gel of purified wild-type and mutant P-loop prototypes. 10 µl of each prototype (100 µM) was mixed with 3 µl of sample buffer and loaded on 16% SDS gel. Samples were run for 1 hr. at 160 V. From left to right: N-$\alpha\beta\alpha$ all GtoE, N-$\alpha\beta\alpha$ $G_3$toE, N-$\alpha\beta\alpha$, ladder, ladder, N-half, N-half $G_3$toA, N-half $G_3$toE, N-half KtoG, ladder. **B.** Comparison of ATP-synthesis activity (expressed as µM.s$^{-1}$) of N-half and its corresponding P-loop mutant prototypes from three independent preparations. **C.** Comparison of ATP-synthesis activity (expressed as µM.s$^{-1}$) of N-$\alpha\beta\alpha$ and its corresponding P-loop mutant prototypes from three independent preparations. Varying concentrations of prototypes (5 µM; black bar, 2.5 µM pink bar; 1.25 µM turquoise bar, 0.6 µM violet bar, 1 mM ADP control; purple bar) were incubated with 1 mM ADP, 0.5 mM

$MgCl_2$ in 50 mM tricine buffer (pH 7.6) for 1 hr. Measurements were performed as described in methods section. Error bars represent standard deviation between two luminescence measurements.

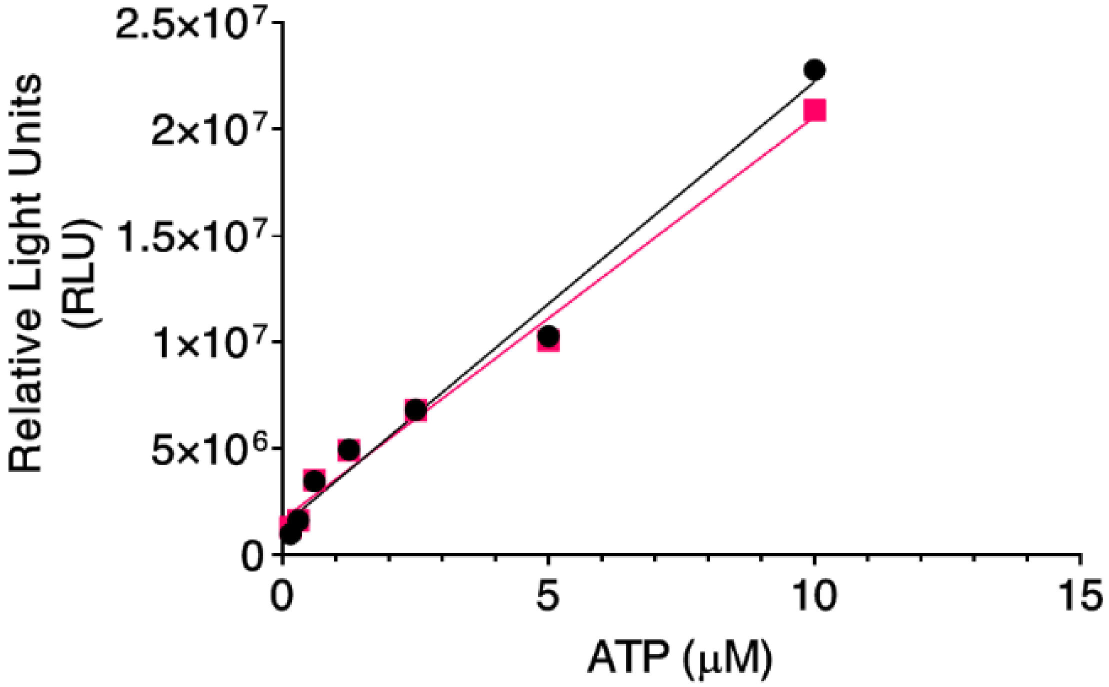


**Figure S12: The ATP-standard curves from freshly prepared luciferase premix (black line) and after 3 hr. of incubation in dark and on ice (pink line).**

**A.**

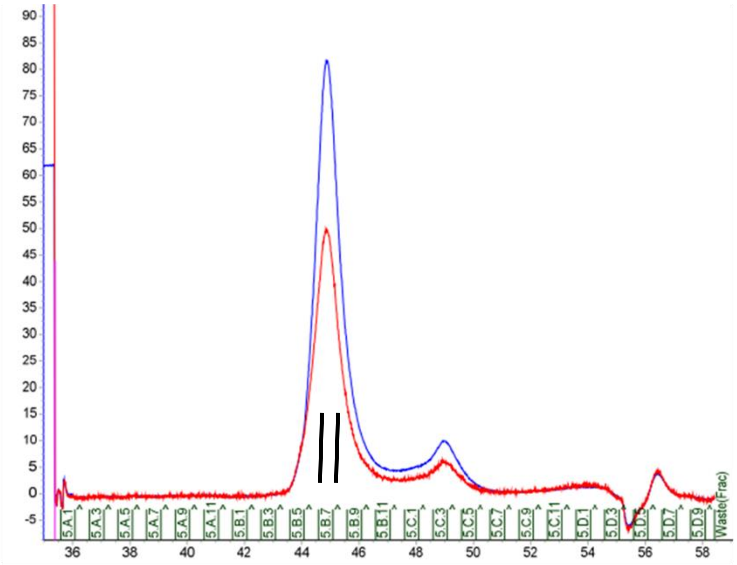


**B.**

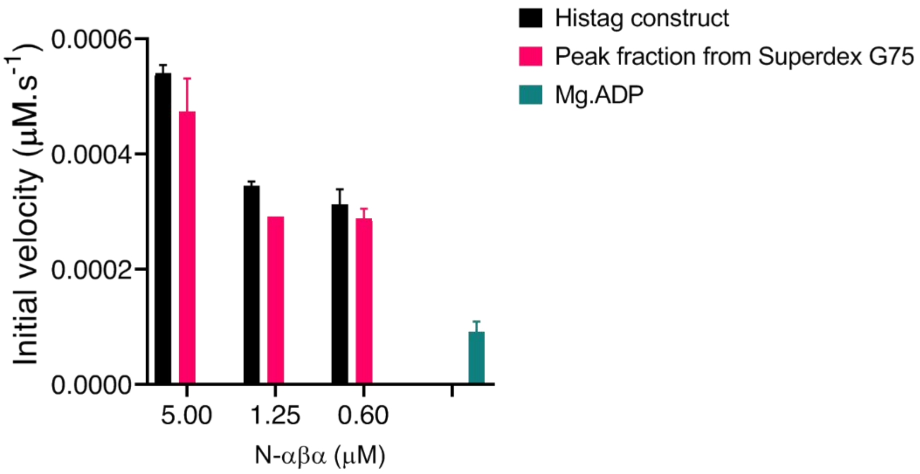


**Figure S13: The purification of N-αβα prototype by size exclusion chromatography. A.** Representative elution chromatogram of N-αβα prototype from Superdex G75 10/300 increase column from two independent expression and purification rounds. The N-αβα prototype, purified by regular guanidine hydrochloride refolding method using NiNTA method (see 'Methods'), was loaded on to the column. 0.5 ml elution fractions were collected. The peak (B7) fraction (indicated by vertical black lines) was collected and subjected to two rounds of dialysis to remove L-arginine. **B.** Comparision of ATP-synthesis activity of N-αβα prototype (purified with

regular method, black bar) with the peak elute (B7 fraction) from size exclusion chromatography (pink bar). Overall, the ATP-synthesis activity of the N-$\alpha\beta\alpha$ prototype does not improve or change upon further purification with the size exclusion chromatography. Therefore, for ease of purification, the constructs were purified using regular, guanidine hydrochloride refolding method using a NiNTA column.

# III. SUPPLEMENTARY TABLES

## Table S1: DNA sequences of P-loop prototypes.

| Protein | Coding DNA sequence |
|---|---|
| Intact prototype (ref. [7,8]) | ATGCGTGTGATTATCGTAATTGTGGGTCCAAGCGGCGCAGG<br>CAAAACCACCCTGCTCGAACTGGCTAAAGAAGCTAAGAAGG<br>AGGTGCCGGATGCTGAGGTTCGCACGGTGACTACTCGTGAT<br>GATGCAAAACGTGTAGCGGAAGAAGCGAAGCGTCGCGGTGT<br>TGATATTGTGGTCATCGTAGGTCCTTCCGGTAGCGGCAAATC<br>TACTCTCGCTACGGAAATCCGTCGCATTATCAAGGAGGCGG<br>GTGCGCGCGACGTTGAGGTCACTACTTCTGAAGAGCTCCGT<br>AAAGCGGCACGTGAAGCTCGTGGCTCCTGGTCTCTCGAGCA<br>CCACCACCACCACCACTGA |
| N-half (ref. [7]) | ATGCGTGTGATTATCGTAATTGTGGGTCCAAGCGGCGCAGG<br>CAAAACCACCCTGCTCGAACTGGCTAAAGAAGCTAAGAAGG<br>AGGTGCCGGATGCTGAGGTTCGCACGGTGACTACTCGTGAT<br>GATGCAAAACGTGTAGCGGAAGAAGCGAAGCGTCGCGGTGT<br>TTGGCTCGAGCACCACCACCACCATCACTGA |
| N-half $G_3$ to A | ATGCGTGTGATTATCGTAATTGTGGGTCCAAGCGGCGCAGC<br>AAAAACCACCCTGCTCGAACTGGCTAAAGAAGCTAAGAAGG<br>AGGTGCCGGATGCTGAGGTTCGCACGGTGACTACTCGTGAT<br>GATGCAAAACGTGTAGCGGAAGAAGCGAAGCGTCGCGGTGT<br>TTGGCTCGAGCACCACCACCACCATCACTGA |
| N-half $G_3$ to E | ATGCGTGTGATTATCGTAATTGTGGGTCCAAGCGGCGCAGA<br>AAAAACCACCCTGCTCGAACTGGCTAAAGAAGCTAAGAAGG<br>AGGTGCCGGATGCTGAGGTTCGCACGGTGACTACTCGTGAT<br>GATGCAAAACGTGTAGCGGAAGAAGCGAAGCGTCGCGGTGT<br>TTGGCTCGAGCACCACCACCACCATCACTGA |
| N-half K to A | ATGCGTGTGATTATCGTAATTGTGGGTCCAAGCGGCGCAGG<br>CGCAACCACCCTGCTCGAACTGGCTAAAGAAGCTAAGAAGG<br>AGGTGCCGGATGCTGAGGTTCGCACGGTGACTACTCGTGAT<br>GATGCAAAACGTGTAGCGGAAGAAGCGAAGCGTCGCGGTGT<br>TTGGCTCGAGCACCACCACCACCATCACTGA |
| N-$\alpha\beta\alpha$ (ref. [7]) | ATGACTCGTGATGATGCAAAACGTGTAGCGGAAGAAGCGAA<br>GCGTCGCGGTGTTGGTAGCGGCCGTGTGATTATCGTAATTG<br>TGGGTCCAAGCGGCGCAGGCAAAACCACCCTGCTCGAACTG<br>GCTAAAGAAGCTAAGAAGGAGGTGTGGCTCGAGCACCACCA<br>CCACCATCACTGA |
| N-$\alpha\beta\alpha$ $G_3$ to E (ref. [7]) | ATGACTCGTGATGATGCAAAACGTGTAGCGGAAGAAGCGAA<br>GCGTCGCGGTGTTGGTAGCGGCCGTGTGATTATCGTAATTG<br>TGGGTCCAAGCGGCGCAGAAAAAACCACCCTGCTCGAACTG<br>GCTAAAGAAGCTAAGAAGGAGGTGTGGCTCGAGCACCACCA<br>CCACCATCACTGA |
| N-$\alpha\beta\alpha$ allG to E | ATGACTCGTGATGATGCAAAACGTGTAGCGGAAGAAGCGAA<br>GCGTCGCGGTGTTGGTAGCGGCCGTGTGATTATCGTAATTG<br>TGGAACCAAGCGAAGCAGAAAAAACCACCCTGCTCGAACTG<br>GCTAAAGAAGCTAAGAAGGAGGTGTGGCTCGAGCACCACCA<br>CCACCATCACTGA |

## Table S2: Amino acid sequences of P-loop prototypes

The P-loop sequence motifs are shown in bold red and mutations to the P-loop are indicated in bold black. All prototypes contained a C-terminal expression tag that included a Trp residue to allow determination of protein concentration by absorbance at 280 nm, and a 6xHis tag for purification (annotated in italics). Molecular weight and theoretical isoelectric point for each construct was calculated from the amino-acid sequence using ExPASy ProtParam tool [9]

| **Protein** | **Amino acid sequence** | **Molecular weight (daltons)** | **Isoelectric point** |
|---|---|---|---|
| Intact prototype (ref. [7,8]) | MRVIIVIV**GPSGAGKT**TLLELAKEAKKEVPDAEVRTVTTRDDAKRVAEEAKRRGVDIVVIV**GPSGSGKS**TLATEIRRIIKEAGARDVEVTTSEELRKAAREARGS*WSLEHHHHHH* | 12561.32 | 9.45 |
| N-half (ref. [7]) | MRVIIVIV**GPSGAGKT**TLLELAKEAKKEVPDAEVRTVTTRDDAKRVAEEAKRRGV*WLEHHHHHH* | 7211.28 | 9.30 |
| N-half $G_3$ to A | MRVIIVIV**GPSGAAKT**TLLELAKEAKKEVPDAEVRTVTTRDDAKRVAEEAKRRGV*WLEHHHHHH* | 7225.31 | 9.30 |
| N-half $G_3$ to E | MRVIIVIV**GPSGAEKT**TLLELAKEAKKEVPDAEVRTVTTRDDAKRVAEEAKRRGV*WLEHHHHHH* | 7283.35 | 8.21 |
| N-half K to A | MRVIIVIV**GPSGAGAT**TLLELAKEAKKEVPDAEVRTVTTRDDAKRVAEEAKRRGV*WLEHHHHHH* | 7154.19 | 8.25 |
| N-αβα (ref. [7]) | MTRDDAKRVAEEAKRRGVGSGRVIIVIV**GPSGAGKT**TLLELAKEAKKEV*WLEHHHHHH* | 6443.40 | 9.60 |
| N-αβα $G_3$ to E (ref. [7]) | MTRDDAKRVAEEAKRRGVGSGRVIIVIV**GPSGAEKT**TLLELAKEAKKEV*WLEHHHHHH* | 6515.47 | 9.30 |
| N-αβα allG to E | MTRDDAKRVAEEAKRRGVGSGRVIIVIV**EPSEAEKT**TLLELAKEAKKEV*WLEHHHHHH* | 6659.59 | 7.30 |

## Table S3: The rates of the ATP-synthesis activity of N-half prototype

| 4 μM N-half prototype | | |
|---|---|---|
| **Time interval (minutes)** | **Rate (ATP.min$^{-1}$)** | **$R^2$** |
| **0-5** | 0.18 (0.03) | 0.98 |
| **5-30** | 0.10 (0.02) | 0.98 |
| **30-100** | 0.06 (0.01) | 0.82 |
| | | |
| **1 μM N-half prototype** | | |
| **Time interval (minutes)** | **Rate (ATP.min$^{-1}$)** | **$R^2$** |
| **0-5** | 0.05 (0.01) | 0.71 |
| **5-30** | 0.03 (0.0004) | 0.97 |
| **30-100** | 0.02 (0.0009) | 0.95 |

Reactions were carried out in presence of 0.5 mM $MgCl_2$ at 37°C with 1 mM ADP. Relative light units were converted to ATP using ATP standard curves as described earlier. Data from various time intervals (0-5, 5-30, 30-100 min.) were fit to simple linear regression equation using GraphPad Prism (8.3.0). Values in parenthesis represents SEM from three independent experiments.

# IV. REFERENCES FOR SUPPLEMENTARY INFORMATION


1. Gupta RK, Benovic JL. Magnetic resonance studies of the interaction of divalent metal cations with 2,3-bisphosphoglycerate. *Biochem Biophys Res Commun*. 1978;84(1):130-137. doi:https://doi.org/10.1016/0006-291X(78)90273-5
2. Blair JM. Magnesium, Potassium, and the Adenylate Kinase Equilibrium. *Eur J Biochem*. 1970;13(2):384-390.
3. Chinopoulos C, Vajda S, Csanády L, Mándi M, Mathe K, Adam-Vizi V. A novel kinetic assay of mitochondrial ATP-ADP exchange rate mediated by the ANT. *Biophys J*. 2009;96(6):2490-2504. doi:10.1016/j.bpj.2008.12.3915
4. Gout E, Rébeillé F, Douce R, Bligny R. Interplay of Mg2+, ADP, and ATP in the cytosol and mitochondria: Unravelling the role of Mg2+ in cell respiration. *Proc Natl Acad Sci U S A*. 2014;111(43):E4560-E4567. doi:10.1073/pnas.1406251111
5. K. BURTON. Formation constants for the complexes of orthophosphate with magnesium and hydrogen ions. *Biochem J*. 1959;71(2):388-395. doi:10.1016/0039-9140(85)80027-8
6. Sonn-Segev A, Belacic K, Bodrug T, et al. Quantifying the heterogeneity of macromolecular machines by mass photometry. *Nat Commun*. 2020;11(1):1772. doi:10.1038/s41467-020-15642-w
7. Vyas P, Trofimyuk O, Longo LM, Deshmukh FK, Sharon M, Tawfik DS. Helicase-Like Functions in Phosphate Loop Containing Beta-Alpha Polypeptides. *Proc Natl Acad Sci*. 2021;118(16). doi:10.1101/2020.07.30.228619
8. Romero Romero ML, Yang F, Lin Y-R, et al. Simple yet functional phosphate-loop proteins. *Proc Natl Acad Sci*. 2018;115(51):E11943 LP-E11950. doi:10.1073/pnas.1812400115
9. Gasteiger E, Hoogland C, Gattiker A, Wilkins MR, Appel RD, Bairoch A. Protein identification and analysis tools on the ExPASy server. In: *The Proteomics Protocols Handbook*. Springer; 2005:571-607.
